# Distinct Single Amino Acid Replacements in the Control of Virulence Regulator Protein Differentially Impact Streptococcal Pathogenesis

**DOI:** 10.1371/journal.ppat.1002311

**Published:** 2011-10-20

**Authors:** Nicola Horstmann, Pranoti Sahasrabhojane, Bryce Suber, Muthiah Kumaraswami, Randall J. Olsen, Anthony Flores, James M. Musser, Richard G. Brennan, Samuel A. Shelburne

**Affiliations:** 1 Department of Biochemistry and Molecular Biology, MD Anderson Cancer Center, Houston, Texas, United States of America; 2 Department of Infectious Diseases, MD Anderson Cancer Center, Houston, Texas, United States of America; 3 Center for Molecular and Translational Human Infectious Diseases Research, The Methodist Hospital Research Institute, and Department of Pathology and Genomic Medicine, The Methodist Hospital, Houston, Texas, United States of America; 4 Department of Pediatrics, Baylor College of Medicine, Houston, Texas, United States of America; Children's Hospital Boston, United States of America

## Abstract

Sequencing of invasive strains of group A streptococci (GAS) has revealed a diverse array of single nucleotide polymorphisms in the gene encoding the control of virulence regulator (CovR) protein. However, there is limited information regarding the molecular mechanisms by which CovR single amino acid replacements impact GAS pathogenesis. The crystal structure of the CovR C-terminal DNA-binding domain was determined to 1.50 Å resolution and revealed a three-stranded β-sheet followed by a winged helix-turn-helix DNA binding motif. Modeling of the CovR protein-DNA complex indicated that CovR single amino acid replacements observed in clinical GAS isolates could directly alter protein-DNA interaction and impact protein structure. Isoallelic GAS strains that varied by a single amino acid replacement in the CovR DNA binding domain had significantly different transcriptomes compared to wild-type and to each other. Similarly, distinct recombinant CovR variants had differential binding affinity for DNA from the promoter regions of several virulence factor-encoding genes. Finally, mice that were challenged with GAS CovR isoallelic strains had significantly different survival times, which correlated with the transcriptome and protein-DNA binding studies. Taken together, these data provide structural and functional insights into the critical and distinct effects of variation in the CovR protein on GAS pathogenesis.

## Author Summary

Group A *Streptococcus* (GAS) causes a variety of human infections including invasive disease that can often be deadly. GAS strains that cause serious infections may have alterations in the amino acid sequence of the control of virulence regulator (CovR) protein, but mechanisms by which changes in the CovR protein influence GAS disease are not understood. We determined the crystal structure of the CovR DNA binding region and found that alterations in the CovR protein observed in clinical, invasive GAS isolates are likely to disrupt CovR-DNA interaction and overall CovR structure. In accord with the structural data, CovR proteins with a single amino acid change had distinctly different binding affinities for various GAS virulence-factor encoding genes. Similarly, GAS strains that differed by only the presence of a single CovR amino acid change had distinct gene expression profiles. Finally, mice that were challenged with GAS strains that differed by only a single CovR amino acid replacement had significantly different survival times consistent with the idea that alterations in the CovR protein are a key determinant of clinical outcomes in GAS human infections. These findings provide mechanistic insights into how subtle genetic differences can profoundly impact the severity of bacterial infections.

## Introduction

Precise regulation of virulence factor-encoding gene expression is critical to the pathogenesis of a diverse array of bacteria that infect humans [1], [2], [3]. Thus, it is not surprising that bacteria possess numerous systems for carefully controlling the expression of virulence factor-encoding genes ranging from alternative sigma factors to small RNAs to two-component gene regulatory systems (TCS) [4], [5], [6]. TCS consist of a membrane-embedded histidine kinase that responds to environmental stimuli by altering the phosphorylation status of its cognate response regulator protein thereby affecting the regulator's ability to bind DNA and alter gene expression [7]. Thus, TCS act as an efficient mechanism to directly link alterations in the external environment to gene expression, and therefore are critical to the infectivity of numerous major bacterial pathogens [8], [9], [10].

Group A *Streptococcus* (GAS) causes a diverse array of infections in humans ranging from colonization and uncomplicated pharyngeal and skin infections to necrotizing fasciitis and toxic shock-like syndromes [11]. GAS has long served as a model for understanding the molecular basis of microbial pathogenesis from the standpoints of both virulence factor content and virulence factor regulation [12], [13]. One of the key GAS transcription factors is the control of virulence regulator (CovR), a member of the OmpR/PhoB regulator family [14], [15]. CovR is the response regulator protein of the CovRS TCS and appears to function mainly as a negative regulator by binding to AT-rich DNA regions [14]. The mechanism by which CovR binds DNA appears to differ for various promoters of GAS virulence factor-encoding genes ranging from high-affinity for a single DNA binding site to cooperative binding along long stretches of promoter region DNA [14], [16], [17].

GAS strains in which CovR has been inactivated are hypervirulent in mice [18]. Moreover, variation in CovR amino acid content has been identified in GAS strains recovered from humans with invasive infections indicating that alteration in the CovR protein impacts streptococcal virulence [19], [20]. Also, whole-genome analysis of invasive GAS strains has found significant elevation in the number of single-nucleotide polymorphisms in the *covR* gene relative to the remainder of the GAS genome [21]. However, although CovR variation has been well-recognized, insight into the functional and clinical impact of CovR amino acid residue replacements has been restricted by the absence of CovR structural data and the limited study of GAS strains that differ from each other by only a single amino acid in CovR [22]. Herein we used a combination of structural, biochemical, and genetic analyses to determine the consequences of clinically occurring single amino replacements in the CovR DNA binding domain. Our data provide functional insight into how distinct CovR single amino replacements result in differential effects on streptococcal virulence thereby extending understanding of the genetic underpinnings of microbial pathogenesis.

## Results

### Definition of a wild-type CovR and description of published CovR variants

Whole genome sequencing of 301 serotype M3 GAS strains recently showed a predominant CovR amino acid sequence which is identical to the most common CovR sequence identified in 223 strains of various GAS M protein serotypes analyzed in a separate investigation [23], [24]. Thus we will designate this CovR sequence, which is present in the fully sequenced serotype M3 strain MGAS10870, as wild-type CovR for the remainder of this study [21]. Analysis of published manuscripts and CovR sequences deposited at the NCBI revealed that 36 additional distinct amino acid variants of CovR have been described among clinical GAS isolates (Table 1). Based on homology with proteins of known structure, CovR can be divided into an N-terminal receiver domain (amino acids residues 1-121 which include the regulatory phosphorylation site D53) and a C-terminal DNA binding domain (amino acids 134-228) connected by a flexible linker [14], [25]. Single amino acid replacements in CovR occurring in clinical GAS isolates have been observed in the putative receiver and DNA binding domains (Table 1). Each particular CovR amino acid replacement has occurred in a single clinical isolate except for the R94C, R118C, S154P, and Q216P replacements, each of which have been identified in two distinct strains (Table 1).

**Table 1:** Previously reported amino acid replacements in CovR in clinical GAS isolates.

Amino acid replacement	Putative domain of replacement^a^	M protein serotype	Reference
E9V	Receiver	M3	[21]
D10G	Receiver	M11	[24]
D10Y	Receiver	M89	[24]
H24P, E25D	Receiver/receiver	M1	[64]
E26V	Receiver	M3	[21]
I30F	Receiver	M81	[24]
V33G, E40K	Receiver/receiver	M49	[54]
D53E	Receiver	M1	[24]
D53N	Receiver	M4	[24]
D60Y	Receiver	M3	[24]
G61S	Receiver	M1	[65]
R66H	Receiver	M3	[23]
R67C	Receiver	M3	[23]
T80I	Receiver	M3	[23]
M86V	Receiver	Not reported	[19]
R94C	Receiver	Not reported, M89	[19], [24]
G95S	Receiver	M58	[24]
A96V	Receiver	M1	[24]
K102R	Receiver	M3	[66]
A111V	Receiver	M81	[42]
A111D	Receiver	M1	[24]
I116T, S154P	Receiver/DNA binding	M18	[67]
R118C	Receiver	M3, M3	[21], [24]
R118S	Receiver	M1, M81	[24]
R119C	Receiver	M3	[21]
R119L	Receiver	M3	[24]
R119S	Receiver	M1	[24]
R144C	DNA binding	M1	[19]
S154P	DNA binding	M18	[24]
R158C	DNA binding	M1	[65]
W184C	DNA binding	M3	[24]
N193H	DNA binding	M2	[68]
N193I	DNA binding	M3	[21]
R203S	DNA binding	Not reported	[19]
Q216P	DNA binding	M3, M3	[20], [23]
G222V	DNA binding	M1	[65]

a Domain boundaries based on homology with PrrA from *Mycobacterium tuberculosis* [25].

### The crystal structure of the CovR DNA-binding domain

**Figure 1:**
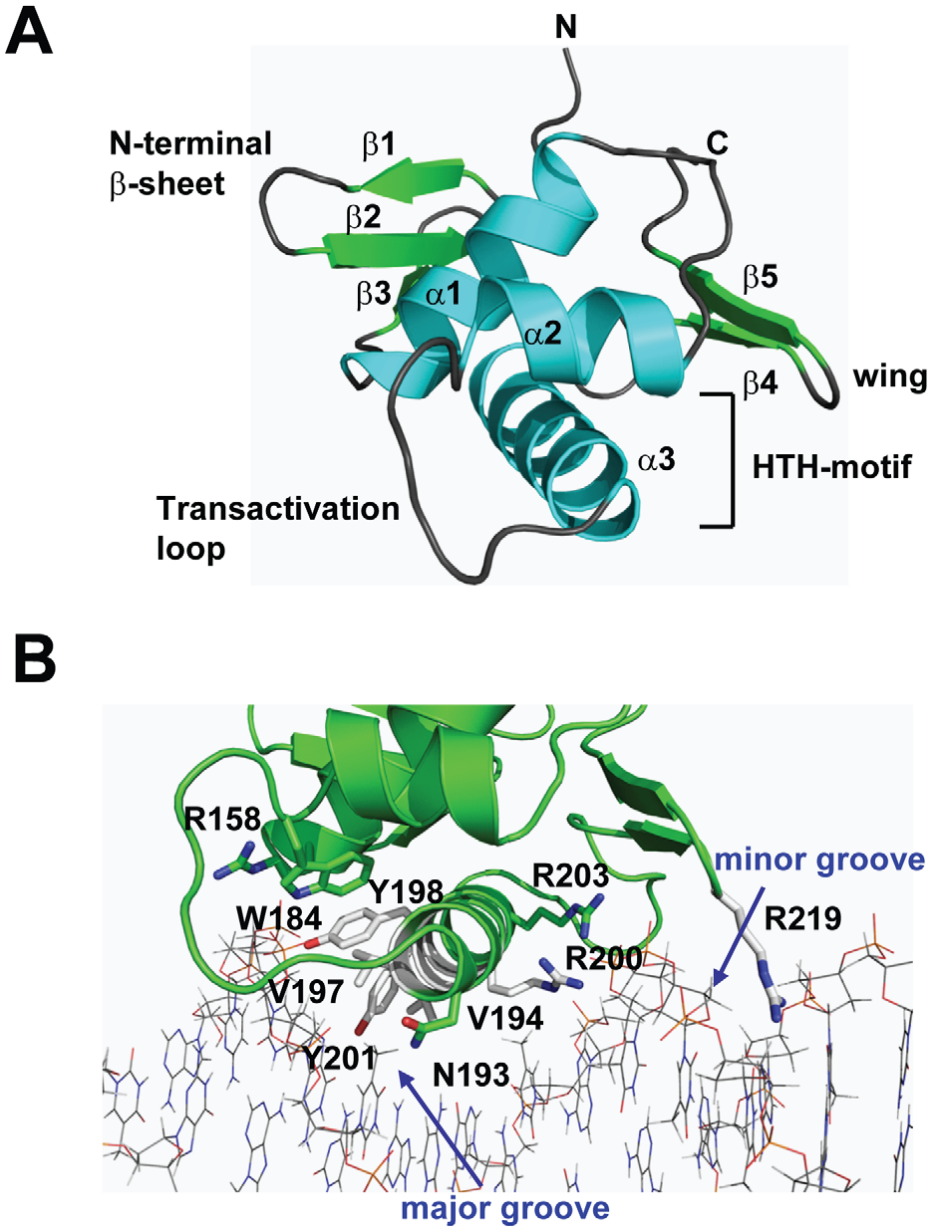
Structure of CovR_CD_. (A) Ribbon diagram of the 1.50 Å resolution crystal structure of CovR_CD_. Amino (N)- and carboxy (C)-termini are labeled. Secondary structure elements are labeled and colored in cyan (α helices), green (β sheets) and gray (loops), respectively. The α helices 2 and 3 constitute the helix-turn-helix motif with α3 as recognition helix. The C-terminal β hairpin forms the wing. (B) Model of the CovR_CD_–DNA complex. The CovR_CD_ crystal structure (shown in green) was modeled onto the 23-bp *pho* Box (colored in gray), which was taken from the crystal structure of the PhoB-DNA complex (PDB ID code 1GXP). Amino acid side chains predicted to interact directly with the phosphate backbone in the major or minor groove of the DNA are shown as sticks. Amino acid residues previously identified as being altered in clinical GAS isolates are shown in green whereas residues not identified as being altered are shown in white.

To gain insight into the structural and functional consequences of CovR single amino acid replacements, we carried out *de novo* crystallization experiments on CovR. Despite extensive effort, we were unable to obtain diffraction quality crystals of the full-length protein. It is known that obtaining full-length crystal structures of OmpR/PhoB family members is difficult due to interdomain flexibility [26]. Therefore we determined the crystal structure of the CovR DNA-binding domain (CovR_CD_ amino acids 134 -228). The structure was solved by the multiple anomalous dispersion (MAD) method [27] and refined to 1.50 Å resolution (Figure 1A). Selected data collection, processing, phasing, and refinement statistics are given in Table 2. The final model is comprised of 95 residues and has the topology: β1(138-140)-β2(144-148)-β3(151-153)-α1(157-168)-α2(176-183)-α3(192-206)-β4(216-218)-β5(221-224) (residues in parentheses). The structure of CovR_CD_ is characterized by a classical helix-turn-helix (HTH) motif (α2-α3) that is flanked on either side by antiparallel β-sheets and buttressed by helix α1 (Figure 1A). The HTH-motif together with the C-terminal β-hairpin forms the canonical winged HTH (wHTH) DNA-binding motif, which is characteristic of members of OmpR/PhoB family of transcription regulators [15]. Consistent with its homology to OmpR/PhoB family members, structural analysis of CovR_CD_ using DALI [28] identified the *Staphylococcus aureus* protein WalR (41% sequence identity to CovR, Z-scores 15.5, root mean square deviation (rmsd)  =  1.8 Å for 94 amino acids) as well as the *Escherichia coli* proteins PhoP and PhoB (32 and 39% sequence identity to CovR, Z = 14.8 and 13.7, rmsd  =  1.6 and 1.5 Å, for 94 and 91 amino acids, respectively) as the closest structural homologues. Structural differences between CovR_CD_ and its structural homologues are mainly present in the N-terminal β-sheet and flexible loops whereas the isolated winged helix-turn-helix motif overlays with ∼0.9 Å rmsd.

**Table 2:** Selected crystallographic data and statistics.

*Data collection and phasing*			
Space group	P2_1_		
Cell dimensions (Å)	a = 30.5	α = 90.0	
	b = 36.7	β = 94.6	
	c = 38.9	γ = 90.0	
Wavelength (λ)	0.9795	1.0199	0.9797
Resolution (Å)	38.82-1.50	38.83-1.50	38.83-1.50
	(1.50-1.58)^b^	(1.50-1.58)	(1.50-1.58)
R_sym_ a	0.054 (0.151)	0.057 (0.119)	0.051 (0.130)
Mean ((I)/σ (I))	21.1 (10.1)	22.5 (12.3)	22.1 (11.1)
Total reflections (#)	83773 (12151)	83548	83729 (12197)
Unique reflections (#)	13145 (1867)	13078	13150 (1876)
Completeness (%)	95.2 (93.7)	94.8 (93.4)	95.2 (93.8)
Multiplicity	6.4 (6.5)	6.4	6.4 (6.5)
Selenium sites	4		
Overall figure of merit c	0.63		
*Refinement statistics*			
Resolution range (Å)	19.5 - 1.50		
R_work_/R_free_ (%)^d^	21.6/23.6		
*Atoms (#)*			
Protein	800		
Solvent	107		
B factors (Å^2^)	53.6		
*RMSD*			
Bond lengths (Å)	0.005		
Bond angles (°)	1.25		
*Ramachandran analysis*			
Ramachandran favored	96.8%		
Ramachandran allowed	3.2%		
Ramachandran outliers (%)	0.00%		

a *R*_sym_  =  ∑∑|I*_hkl(j_*_)_ −I*_hkl_*| / ∑∑I*_hkl_*, where I*_hkl(j)_* is the observed intensity and I*_hkl_* is the final average intensity value.

b Values in parentheses are for the highest resolution shell.

c Figure of Merit  =  <|∑ *P*(*α*)e*^iα^*/∑ *P*(*α*)>, where α is the phase and *P*(*α*) is the phase probability distribution.

d *R*_work_  =  ∑||*F*_obs_| − |*F*_calc_||/∑|*F*_obs_| and *R*_free_  =  ∑||*F*_obs_| − |*F*_calc_||/∑|*F*_obs_|, where all reflections belong to a test set of 5% randomly selected reflections.

### Prediction of functional consequences of single amino acid replacements in the CovR DNA binding domain

Whole-genome sequencing data has indicated that GAS is under evolutionary pressure to vary CovR amino acid composition [21]. Thus, we next sought to analyze the potential functional consequences of single amino replacements in the CovR_CD_. To this end, we modeled the CovR_CD_-DNA complex based on the crystal structure of PhoB_CD_ bound to *pho*-box DNA (PDB code: 1GXP) [29] (Figure 1B). The crystal structure of PhoB_CD_ bound to its cognate DNA is the only protein-DNA complex structure available for the OmpR/PhoB family. Consistent with the modeling data having *in vivo* relevance, the recognition helix α3 and wing of CovR can be docked onto the major and minor grooves of the cognate DNA sequence, respectively, without steric clash (Figure 1B). In this conformation several surface exposed, mainly positively charged, amino acid residues can be positioned favorably to engage in hydrogen bonds and nonspecific polar interactions with the phosphate backbone of the DNA major groove. Such examples include R158 in the first turn of α1, W184 located on the activation loop that connects α2 and α3, as well as recognition helix residues N193, Y198, Y201, R200, and R203. Moreover, the hydrophobic residues V194 and V197 in the recognition helix are positioned to make van der Waals contacts with the methyl group of the thymine bases present in AT-rich CovR DNA binding sites (Figure 1B). In this conformation, wing residue R219 can insert into the minor groove and is likely to interact with the bases and the negatively charged sugar-phosphate backbone.

**Figure 2:**
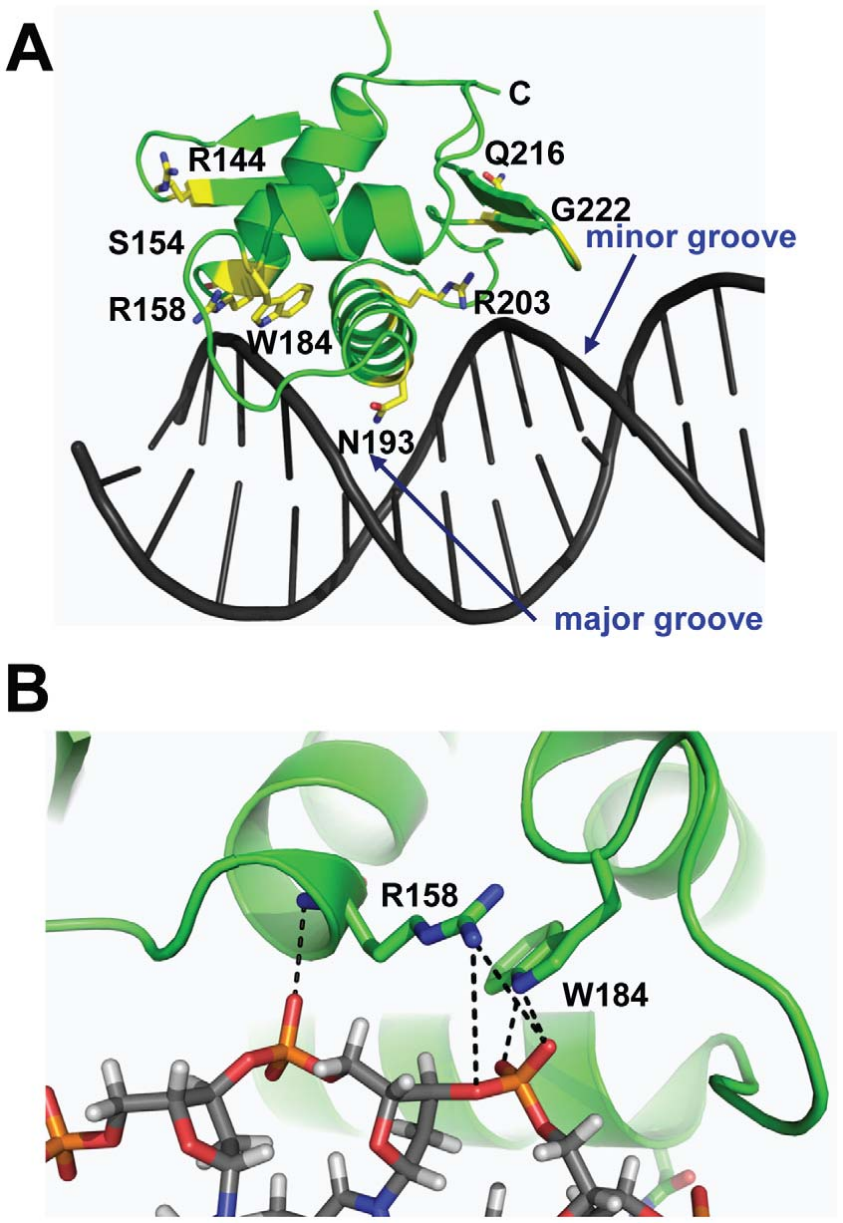
Location of CovR_CD_ residues altered in clinical GAS isolates. The CovR_CD_ was modelled onto DNA as described in Figure 1. (A) Mapping of all CovR_CD_ residues altered in clinical GAS isolates with affected residues depicted in yellow. (B) Close-up view of potential interactions of R158 and W184 with the DNA sugar-phosphate backbone.

By combining the DNA-bound CovR_CD_ modeling and sequencing data (Table 1) we can postulate potential functional effects of amino acid residue replacements in the CovR DNA binding domain. For example, SNPs resulting in the amino acid exchanges R158C, W184C, N193H, N193I, and R203S likely interrupt direct contacts with the DNA (Figure 2A, 2B). In contrast, R144 is located on β2, which is distal to the DNA, and therefore unlikely to be involved in direct DNA contacts. Two proline substitutions (S154P and Q216P) have been observed in clinical GAS isolates (Table 1). These mutations occur at solvent exposed sites with S154P in a loop structure and Q216P at the beginning of wing β4 (Figure 2A). The S154P replacement may result in a local perturbation of the structure of the C-terminus of α1, which impinges upon the activation loop, thereby affecting the position of the HTH motif. The variant CovR protein Q216P has been shown previously to have a DNA binding defect for a single CovR-regulated promoter [20]. Glycine 222 is located on β5 of the wing (Figure 2A). A mutation of this residue to any other would affect CovR structural integrity by producing a steric clash with the peptide backbone of residues M174 and T175. Thus, analysis of CovR_CD_ indicates that observed amino acid replacements are likely to detrimentally impact the binding of CovR to DNA via distinct mechanisms in keeping with the known role of CovR as a negative regulator of virulence factor encoding genes.

### Creation of GAS strains that differ by only a single CovR amino acid replacement

**Figure 3:**
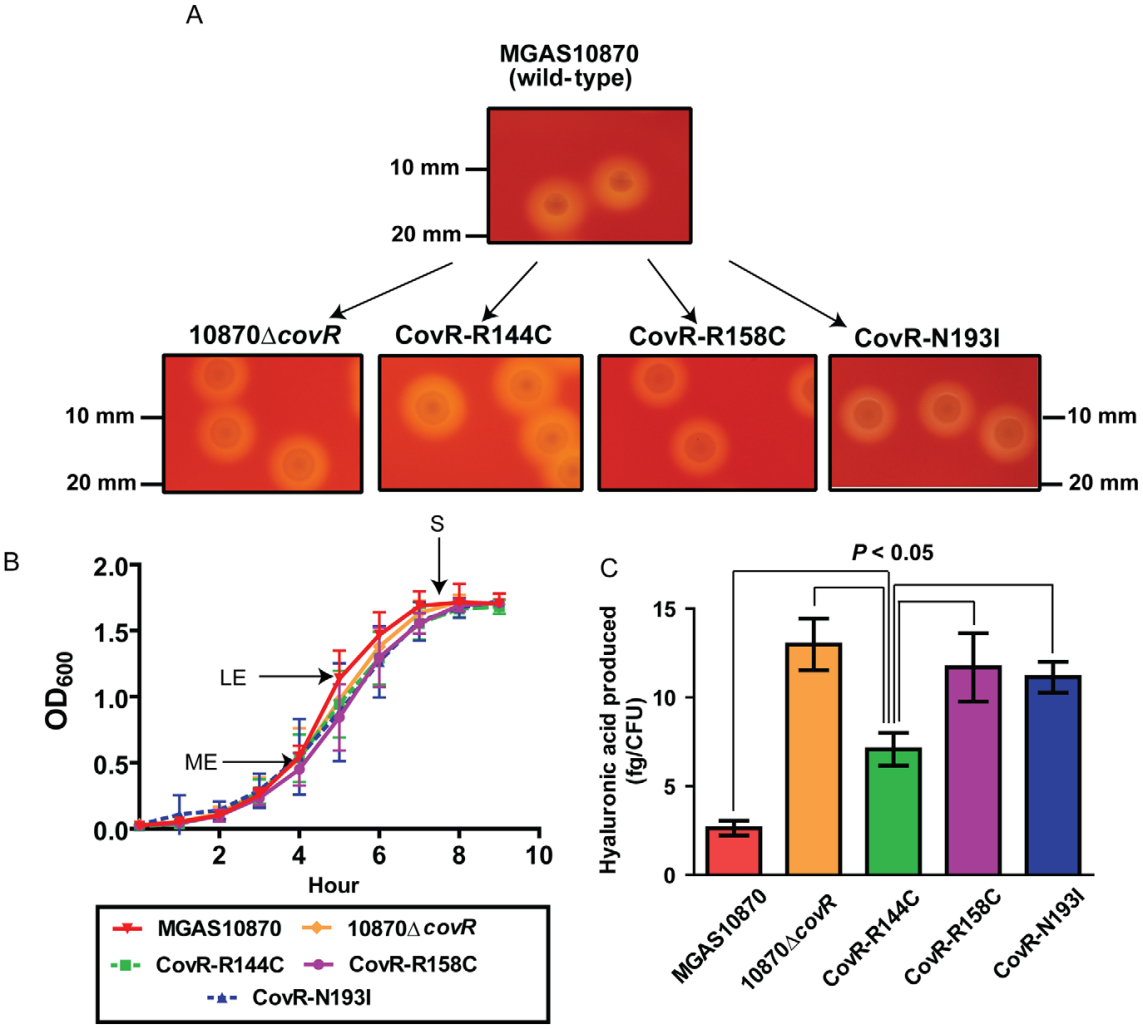
Strain creation schematic and characterization. (A) Insertional inactivation of the CovR-binding domain in strain MGAS10870 was performed to create strain 10870Δ*covR*. Isoallelic exchange introducing SNPs encoding for single amino acid replacements in CovR was used to create strains CovR-R144C, CovR-R158C, and CovR-N193I. Pictures show representative colony morphology following growth on sheep blood agar plates. (B) Indicated strains were grown in a standard laboratory medium with growth measured via spectrophotometry. Growth was done in duplicate on four separate occasions. Data graphed are mean ± standard deviation. Arrows indicate times at which samples were collected for RNA analysis. ME  =  mid-exponential; LE  =  late exponential; S =  stationary. (C) Measurement of cell-wall associated hyaluronic acid (capsule) at LE growth phase. Growth was done in triplicate on three separate occasions with data graphed being mean ± standard deviation. *P* value in panel C is derived from ANOVA followed by Tukey's post-test to account for multiple comparisons.

To gain further insight into the functional consequences of single amino acid replacements in the CovR DNA binding domain, we used isoallelic exchange to create three GAS strains that differed from the parental serotype M3 strain MGAS10870 by only a single CovR amino acid replacement (Table 3, Figure 3A) [30]. We chose to study the R144C, R158C, and N193I substitutions because the R144 residue is not predicted to contact DNA whereas the R158 and N193 residues are predicted to contact distinct parts of the CovR DNA binding site (Figure 2A). We also replaced the entire DNA binding domain of CovR with a kanamycin resistance cassette to create the CovR-inactivated strain 10870Δ*covR*. There was no observable difference in growth rate in a nutrient-rich medium between the wild-type, CovR-inactivated, and CovR-isoallelic strains (Figure 3B). The growth phenotype of GAS on blood agar plates is influenced by hyaluronic acid (HA) capsule production. The level of HA is in part determined by expression of the *hasABC* operon which is negatively regulated through direct binding of CovR to the *hasA* promoter [31]. Compared to wild-type, the CovR-inactivated and CovR-isoallelic strains had a hypermucoid phenotype (Figure 3A). Similarly, cell-associated HA levels were higher in the CovR-inactivated and CovR-isoallelic strains compared to wild-type although HA levels in strain CovR-R144C were lower compared to strains CovR-R158C, CovR-N193I, and 10870Δ*covR* (Figure 3C). These data are consistent with the isogenic strains having increased hyaluronic acid capsule production due to decreased CovR function as would be predicted given the role of CovR as a negative regulator.

**Table 3:** Bacterial strains and plasmids used in this study.

Strain or plasmid	Description	Reference
**Strains**		
MGAS10870	Invasive isolate, serotype M3, CovR wild-type	[21]
10870Δ*covR*	MGAS10870 Δ*covR*::*aphA3*	This study
CovR-R144C	MGAS10870 with *covR* encoding Cys at 144	This study
CovR-R158C	MGAS10870 with *covR* encoding Cys at 158	This study
CovR-N193I	MGAS10870 with *covR* encoding Ile at 193	This study
MGAS315	Invasive isolate, serotype M3	[40]
MGAS5005	Invasive isolate, serotype M1	[41]
Plasmids		
pJL1055	Low-copy number shuttle vector with temperature-sensitive replication in GAS	[69]
pSSCovR-C144	pJL1055 with *covR* encoding Cys at 144	This study
pSSCovR-C158	pJL1055 with *covR* encoding Cys at 158	This study
pSSCovR-I193	pJL1055 with *covR* encoding Ile at 193	This study
pTXB1-CovR	pTXB1 with wild-type *covR*	[50]
pTXB1-CovR-C144	pTXB1 with *covR* encoding Cys at 144	This study
pTXB1-CovR-C158	pTXB1 with *covR* encoding Cys at 158	This study
pTXB1-CovR-I193	pTXB1 with *covR* encoding Ile at 193	This study

### CovR single amino acid replacements result in distinct transcriptomes

**Figure 4:**
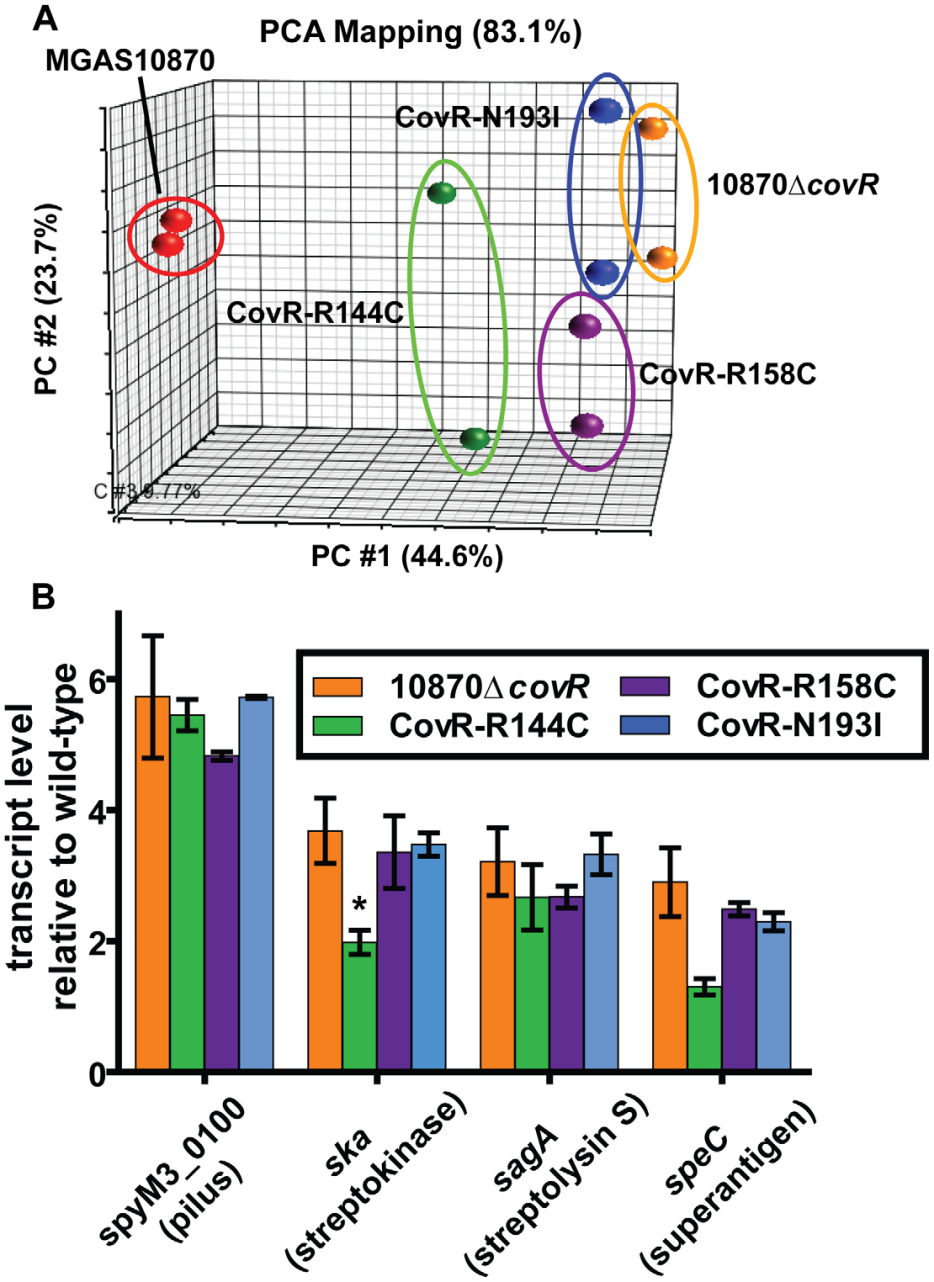
Transcriptome analysis of GAS strains with CovR single amino replacements. Indicated strains were grown to mid-exponential phase in duplicate. (A) Principal components analysis (PCA) showing inter-sample variation for the entire expression microarray data set. The majority of the variation is depicted by location along the horizontal axis. (B) Specific gene transcript levels in indicated strains relative to wild-type. Genes are indicated along with encoded proteins in parentheses. All depicted differences in transcript level compared to wild-type are statistically significant by ANOVA followed by Tukey's post-test to account for multiple comparisons at a *P* value of <0.05 except for *speC* in strain CovR-R144C (indicated by *). Data graphed are mean ± standard deviation for duplicate biological replicates.

As CovR is known to be a global regulatory protein, we next compared the transcriptomes of the wild-type, the CovR-inactivated, and the three CovR-isoallelic strains. Each strain was grown in duplicate to mid-exponential phase (see Figure 3B for time point of RNA analysis) which was chosen to facilitate comparison to other published CovR transcriptome data derived from a serotype M3 strain [32]. Compared to the wild-type strain MGAS10870, we observed distinct transcriptome patterns. Consistent with complete inactivation of CovR, strain 10870Δ*covR* had the highest number of genes with differential transcript levels compared to wild-type (204, or approximately 12% of all genes expressed under the tested conditions) (Table S1). The CovR-isoallelic strains CovR-R144C, CovR-R158C and CovR-N193I had 69, 126, and 156 genes, respectively, with differential transcript levels compared to wild-type (4.2%, 7.8%, and 9.6% of all expressed genes, respectively). In addition to a difference in the absolute numbers of genes affected, we also observed a difference in the magnitude by which transcript levels varied for affected genes among the CovR-inactivated and CovR-isoallelic strains. Compared to wild-type, the mean fold difference in transcript level for differentially expressed genes was 3.01, 2.03, 2.54, and 2.65 for strains 10870Δ*covR*, CovR-144C, CovR-158C, and CovR-N193I, respectively (*P*<0.01 by analysis of variance). Similarly, when differences in the datasets were compared using principal components analysis, the wild-type replicates clustered together and the 10870Δ*covR* replicates were furthest from the wild-type values (Figure 4A). Strains CovR-R158C and CovR-N193I were clustered near strain 10870Δ*covR* whereas the transcriptomes of the CovR-R144C replicates were located between those of the wild-type and the other three strains (Figure 4A). These data are consistent with the R144C replacement having a less severe effect on CovR function compared to the R158C and N193I replacements which is as expected given the key role in DNA binding for R158 and N193 predicted by the structural data (Figure 1B).

Virulence factor encoding genes whose transcript levels were significantly different compared to wild-type in the CovR-inactivated and each of the CovR-isoallelic strains included the operon encoding the GAS pilus that is important for adhesion to eukaryotic cells (spyM3_0098 to spyM3_0102) [33], the gene encoding the plasminogen activating protein streptokinase (Ska) [34], and the operon encoding the cytotoxin streptolysin S (SLS) [35]. The pilus genes have not previously been reported to be directly regulated by CovR whereas *ska* and the SLS-encoding operon are known to be CovR regulated [16], [17]. Compared to wild-type, the pilus operon and SLS-encoding operon were influenced to a similar degree in each of the other four strains whereas the increase in *ska* transcript level was greater in the CovR-inactivated, CovR-R158C, and CovR-N193I strains compared to strain CovR-R144C (Figure 4B). Similarly, the transcript level of the gene encoding the superantigen SpeC was increased compared to wild-type in strains 10870Δ*covR*, CovR-R158C, and CovR-N193I but was not significantly elevated versus wild-type in strain CovR-R144C (Figure 4B). Taken together, we conclude that single amino acid residue replacements in CovR result in transcriptomes that vary in the total number of genes affected as well as in the degree to which transcript levels are affected depending on the particular amino acid replacement.

### Transcript level variation in the CovR isoallelic strains is dependent on growth phase and gene assayed

**Figure 5:**
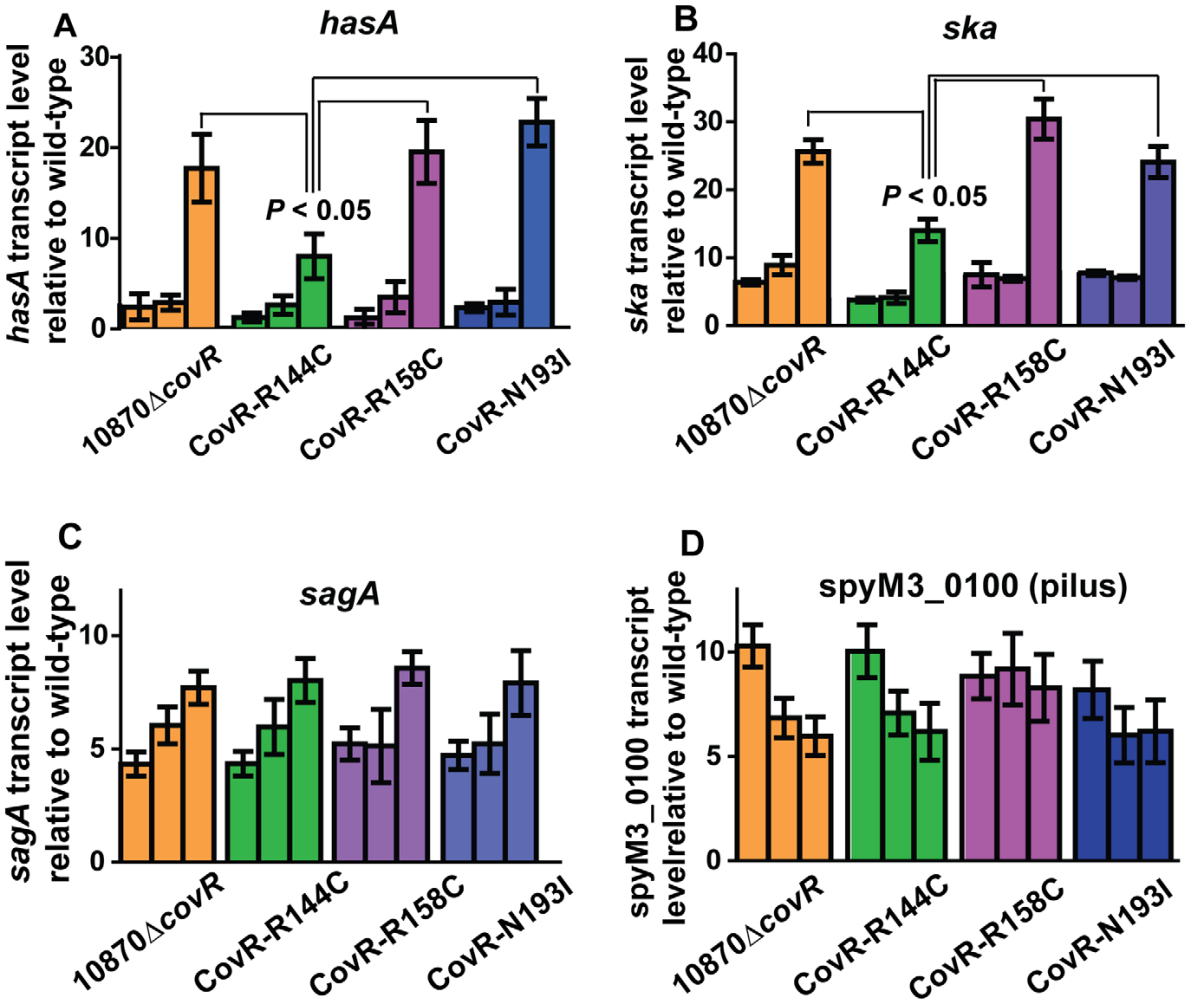
Influence of CovR single amino replacements on transcript level is gene and growth phase dependent. For all panels indicated strains were grown as described in THY, and RNA was harvested, converted to cDNA and analyzed for indicated gene transcript level via TaqMan QRT-PCR. In each panel, there are three bars for each strain corresponding from left-to-right to mid-exponential, late-exponential, and stationary growth phase respectively (see Figure 3 A). Data graphed are mean ± standard deviation of duplicate biologic replicates performed on two separate occasions and analyzed in duplicate (total of 8 data points) of transcript level in the indicated strain relative to the wild-type strain MGAS10870. (A) *hasA* (first gene in capsule operon), (B) *ska* (gene encoding streptokinase), (C) *sagA* (first gene in streptolysin S operon), and (D) spyM3_0100 (gene in pilus operon). *P* values in (A, B) are derived from two-sided Student's *t-*test comparing the transcript level in strain CovR-R144C to each of the other three strains.

The microarray data provide insight into global gene expression at one time point in the growth cycle. Given that CovR influence can vary depending on the growth phase [32], [36], we next used TaqMan QRT-PCR to assay the transcript level of select genes at multiple time points in the growth cycle. In accord with the observed differences in colony morphology and HA production (Figure 3), the CovR-inactivated and CovR-isoallelic strains had significantly increased *hasA* transcript level relative to wild-type, particularly during stationary phase (Figure 5A). However, the increase in *hasA* transcript level was significantly higher in strains 10870Δ*covR*, CovR-R158C, and CovR-N193I compared to strain CovR-R144C consistent with the R144C replacement having a less severe effect on CovR function compared to the R158C and N193I replacements. Similar to the *hasA* data, strain CovR-R144C had a *ska* transcript level that was intermediate between wild-type and the other isoallelic strains (Figure 5B). Thus, for *hasA* and *ska*, the CovR-R158C and N193I replacements had a more significant effect on transcript level compared to the R144C replacement.

For other virulence factor encoding genes, however, the R144C replacement had similar effects to the R158C and N193I replacements. For example, compared to wild-type there were similar increases in the transcript level of *sagA* and the gene encoding for the IL-8 degrading enzyme SpyCEP [37] for the CovR-inactivated and each of the three CovR-isoallelic strains at all time points tested, although the magnitude of increase varied depending on the growth phase (Figure 5C, Figure S1A). We also observed similar increases in the transcript level of the pilus encoding operon genes spyM3_0099 and spyM3_0100 in each of the CovR-inactivated/CovR-isoallelic strains compared to wild-type (Figure S1B, Figure 5D). Finally, the transcript level of the *nra* gene, which encodes a protein known to regulate the pilus operon [38], was similarly increased in each of the CovR-inactivated/CovR-isolallelic strains (Figure S1 C). Taken together, we conclude that distinct single amino acid residue replacements in CovR differentially affect GAS gene transcript levels in a growth phase-dependent and gene-specific fashion.

### Recombinant CovR binds to DNA from the *cpb*/*nra* promoter

**Figure 6:**
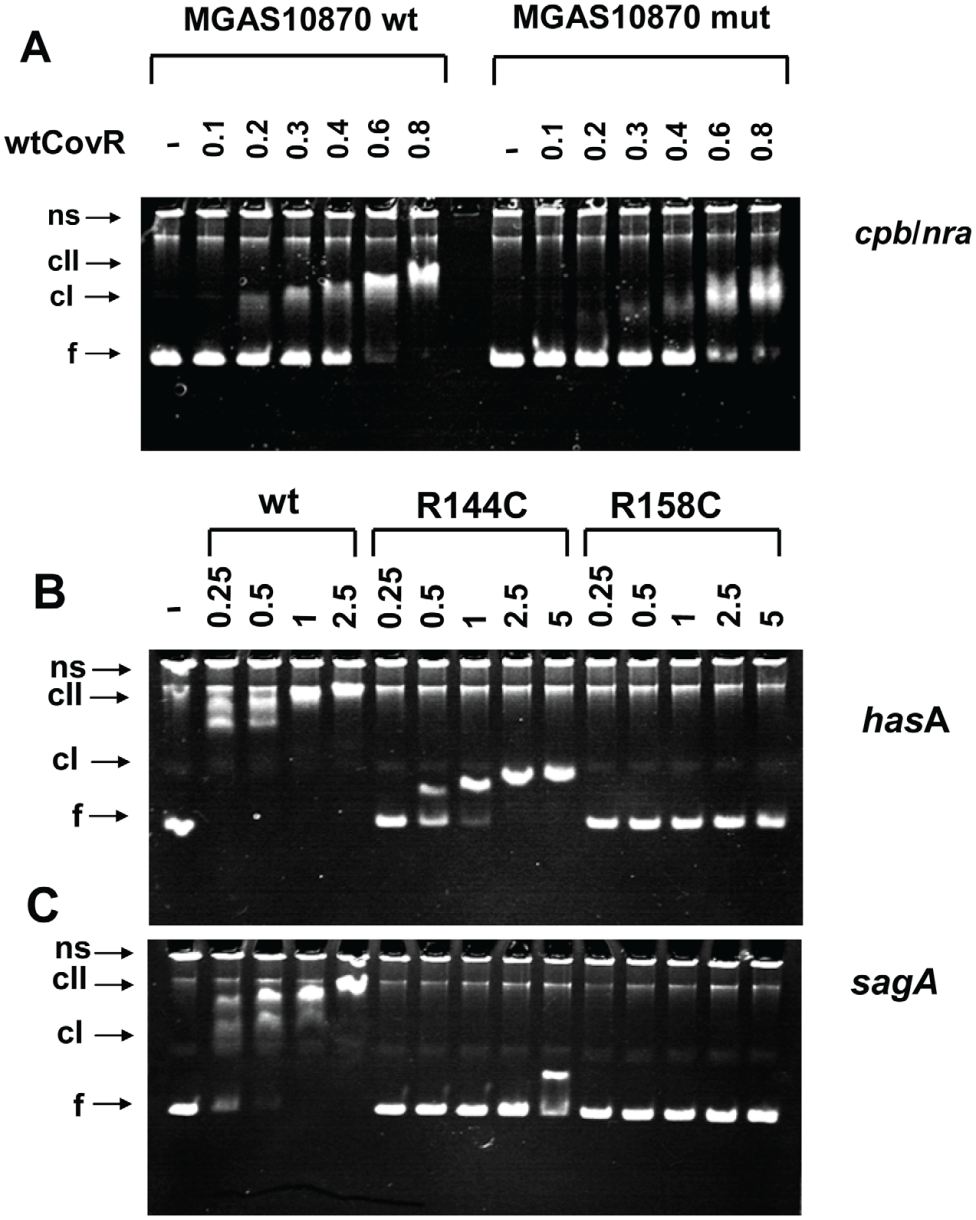
Electromobility shift assays of recombinant CovR DNA binding. (A) Binding of wild-type CovR to DNA from the *cpb*/*nra* (pilus operon) promoter of serotype M3 strain MGAS10870 and DNA in which the ATTARA sites from the *cpb/nra* promoter have been altered (for details see Materials and Methods). (B and C) Effect of amino acid residue replacements in CovR on binding to the promoters of *hasA* (first gene in the hyaluronic acid capsule encoding operon) (B) and *sagA* (first gene in the streptolysin S encoding operon) (C). Increasing concentrations (in µM) of phosphorylated CovR wild type, CovR-R144C, and CovR-R158C were used as indicated. For all panels the samples were incubated at 37°C for 15 min and electrophoresed at 110V for 70 min. The resulting 6% polyacrylamide gel was stained with ethidium bromide. ns, nonspecific DNA; f, non-complexed/free DNA; cI, lower molecular weight complex; cII higher molecular weight complex. Gels shown are representative of identical results obtained on three separate occasions.

Given that CovR has not previously been shown to directly regulate the expression of the pilus operon, we next examined the pilus promoter region from strain MGAS10870 and the fully annotated serotype M3 strains MGAS315 and SSI-1 [39], [40]. In these strains, the initial gene of the pilus operon (*cbp*) is divergently transcribed from the gene encoding the regulator protein Nra (Figure S2). There are five putative CovR consensus binding sites (i.e. ATTARA sequences) in the 430 bp *cbp/nra* intergenic region (Figure S2). Notably, none of these putative CovR binding sites is present in the analogous region in the fully annotated serotype M1 strain MGAS5005 in which CovR inactivation did not affect transcript level of the pilus operon genes [36], [41]. Consistent with this observation, electromobility shift assays (EMSA) verified direct binding of full-length, recombinant CovR to the *cpb/nra* intergenic region from the serotype M3 strains MGAS10870 and MGAS315 (Figure 6A, Figure S3A). Recombinant CovR also bound the *cpb/nra* intergenic region in which the putative CovR binding sites had been mutated as well as the respective region from the serotype M1 strain MGAS5005, albeit with significantly reduced affinity compared to DNA from the serotype M3 strains (Figure 6A, Figure S3A). Together with previous observations, these data indicate that tight binding of CovR to the *cpb/nra* inter-genic region is required to influence pilus operon gene transcript levels.

### DNA affinity of recombinant CovR variants is promoter dependent

Our transcript level data showed that CovR repression of virulence factor expression is relieved to different extents in GAS strains containing distinct SNPs in the *covR* gene (Figure 5). Therefore, we next tested the hypothesis that CovR variants resulting from distinct single amino acid replacements have different DNA binding activities. We were able to obtain high yields of soluble, full-length, recombinant CovR-R144C and CovR-R158C (Figure S4A), but because of solubility issues, CovR-N193I was not further investigated. We compared the binding of the phosphorylated wild-type, R144C, and R158C CovR variants to DNA from the promoters of three known CovR-regulated virulence factor-encoding genes: *hasA, sagA*, and *ska.* In agreement with previous studies, wild-type CovR∼P bound the promoters of *hasA* (Figure 6B), *sagA* (Figure 6C), and *ska* (Figure S3B) at a protein concentration of 0.25-0.5 µM and showed a complete shift at ∼1 µM [16], [17], [31]. CovR-R158C∼P had no detectable DNA binding to all three promoters up to a protein concentration of 5 µM (Figure 6, Figure S3B). In contrast, CovR-R144C ∼P evidenced minimal binding to the *sagA* promoter but did display binding to the promoters of *hasA* and *ska*, albeit with ∼ 4-10 fold lower affinity compared to the wild type (Figure 6, Figure S3B). However, the DNA retardation is much less compared to the wt, indicating that a higher molecular weight complex, as seen for wt CovR∼P, cannot be formed with CovR-R144C∼P (at this concentration). Thus, *in vitro* the DNA binding defect engendered by the CovR-R144C replacement is less severe than the R158C replacement, and the effect of the R144C replacement on DNA binding is different depending on the promoter being studied. These data provide a functional explanation for our finding that the transcriptome of strain CovR-R144C was intermediate between the wild-type and the CovR-R158C strains and that the transcript levels of *hasA* and *ska* were less in strain CovR-R144C compared to strain CovR-158C (Figure 4, Figure 5).

### Analysis of secondary structure of CovR variant proteins

A possible explanation for the impaired *in vitro* DNA binding activity of the CovR variants is that the recombinant variants are improperly folded. Thus, we analyzed the secondary structure of recombinant, full-length wild-type and CovR variant proteins using circular dichroism (CD). The CD spectra for each of the CovR proteins studied showed characteristics of a mixed α/β protein (Figure S4B). The spectra for wild-type CovR and CovR-R144C were identical indicating that the R144C replacement did not result in a structural change in the absence of DNA. However, compared to wild-type, we observed more pronounced minima at 220 nm and 208 nm for CovR-R158C indicating a higher degree of α helical structure (Figure S4B). One possible explanation for this observation is that in the absence of DNA the N-terminus of α1 is less helical when R158 is present due to electrostatic repulsion between the positive dipole of the helix and positive charge of the guanidinium side chain. Alternatively, when C158 is present the sulphydryl side chain of cysteine might show a significant thiolate character and thus interact favorably with the helical dipole. The CD difference between wild-type and CovR-R158C was also observed in the presence of 1 mM TCEP as a reducing agent suggesting that the introduction of cysteine does not induce intermolecular disulphide bond formation thereby altering the secondary structure (unpublished data). Taken together, we conclude that the recombinant wild-type CovR and R144C and R158C variants are folded similarly but that the R158C replacement induces a slightly more helical structure compared to the wild-type protein in the absence of DNA.

### Single amino acid residue replacements in CovR differentially affect GAS virulence

**Figure 7:**
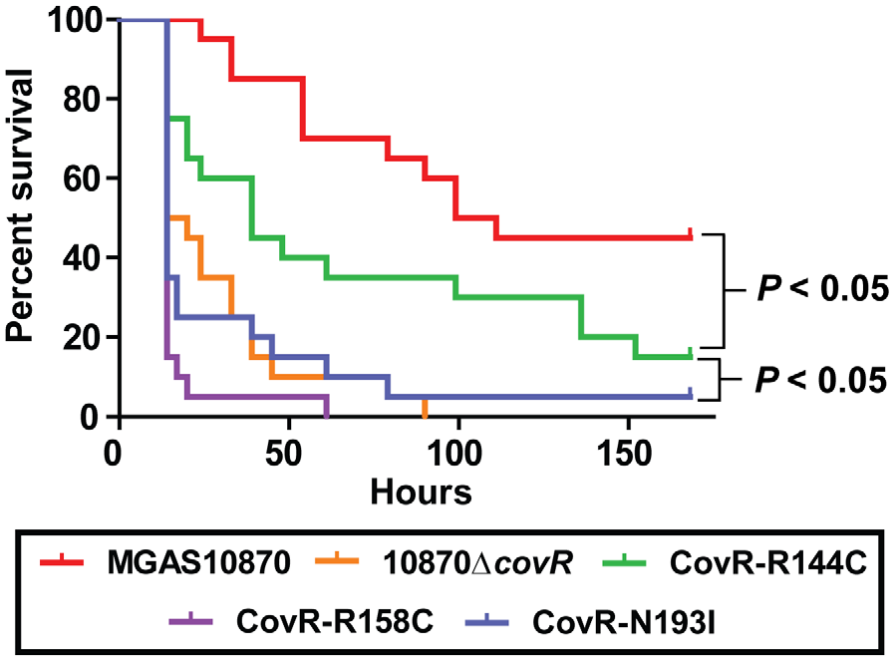
CovR single amino acid replacements result in differential virulence in mice. 20 outbred CD-1 mice were infected intraperitoneally with 1.0×10^7^ CFUs of each indicated strain. Data shown are survival over time with *P* values derived from Kaplan-Meier survival analysis.

In light of our observations regarding the significant effect of CovR variation on global gene expression and protein function, we next tested the hypothesis that GAS strains harboring distinct CovR variants would have differential virulence. To this end, we challenged mice intraperitoneally with 1×10^7^ colony forming units (CFUs) of strain MGAS10870, its CovR-inactivated derivative, and the three CovR isoallelic strains. Each of the tested strains caused some extent of near-mortality in mice (Figure 7). However, the survival times of the mice infected with the five strains were significantly different (*P*<0.0001 by log-rank test). Specifically, the survival time of mice infected with strain MGAS10870 was significantly longer than mice infected with each of the other four strains (Figure 7) (*P*<0.05 for MGAS10870 compared to strain CovR-R144C and *P*<0.01 for strain MGAS10870 compared to either strain 10870Δ*covR*, CovR-R158C, or CovR-N193I). Similarly, the survival time of mice infected with strain CovR-R144C was significantly longer than mice infected with strains 10870Δ*covR*, CovR-R158C, or CovR-N193I (*P*<0.05 for each comparison). There was no significant difference in the survival times among mice infected with strains 10870Δ*covR*, CovR-R158C, or CovR-N193I (*P* = 0.35). Thus, we conclude that CovR single amino replacements occurring in GAS clinical isolates significantly impact strain virulence and that the degree of impact differs depending on the particular amino acid substitution. These findings are in accordance with our transcriptome, QRT-PCR, and DNA binding activities showing a CovR-R144C phenotype intermediate between wild-type and strains 10870Δ*covR*, CovR-R158C, and CovR-N193I.

## Discussion

It is well recognized that patients with severe, invasive GAS disease can be infected with strains that have variation in the CovR protein (Table 1) [42]. Thus, CovR stands as a paradigm for studying how small-scale genetic changes in infecting microbes can influence disease manifestations. Despite extensive previous investigation of CovR function, however, prior to the work described herein knowledge regarding mechanisms by which single amino acid variation in CovR influences GAS virulence was limited [20], [22].

A key finding of this work was that GAS strains that contain different single amino acid residue replacements in CovR have distinct transcriptomes and virulence profiles from strains with a wild-type CovR and from each other (Figure 4A, Figure 7). Specifically, we found that the transcriptome of strain CovR-R144C was different from wild-type and from strains that contained the R158C and N193I amino acid replacements (Figure 4A). Previous studies have examined the effects of a single CovR amino acid replacement and thus could not compare the effects of replacements at distinct sites [20], [22]. Studies of other microbial proteins that examined effects of single amino replacements found a binary effect on virulence in which particular amino acid replacements either did or did not affect virulence [30], [43]. Our data show that the effect of CovR single amino acid replacements on virulence occurs on a graded scale with particular replacements (e.g. R144C) causing a significant difference compared to wild-type but having a less pronounced effect compared to other replacements (e.g. R158C and N193I). Given the large number of CovR amino acid variations yet to be investigated it is likely that similar differential effects will be observed depending on the particular amino acid involved.

The differential effects of the R144C, R158C, and N193I replacements can be explained by a combination of our structural and functional data. The R158C and N193I replacements resulted in a CovR-inactivated phenotype in terms of gene expression (Figs. 4 and 5) and virulence (Figure 7) consistent with the complete lack of DNA binding observed for recombinant CovR-R158C (Figure 6). Previous mutagenesis studies of OmpR/PhoB family proteins have identified amino acids in analogous positions to CovR R158 and N193 as being important for protein function [44], [45], and our structural data suggest a direct role for these two residues in CovR DNA binding (Figure 2). Conversely, the R144C replacement resulted in a gene expression, DNA-binding, and virulence profile that was intermediate between wild-type and the CovR-inactivated strain. Given the distance from DNA of the R144 residue (Figure 2A), how the R144C replacement affects CovR function is not clear, and the equivalent residue has not been typically identified as critical to the function of OmpR/PhoB family members. Interestingly, introducing a cysteine at the equivalent residue in *E. coli* OmpR, R150, also resulted in a differential effect on gene expression as there was decreased OmpR-mediated regulation of the *ompC* but not the *ompF* gene [46]. In addition to the previously established head-to-tail conformation, crosslinking experiments indicated a head-to-head arrangement of OmpR dimers in which R150 is part of the intermolecular dimerization interface formed by opposing β-sheets [46]. Thus, the R150C substitution is predicted to affect the function of OmpR dimers arranged in a head-to-head, but not head-to-tail, conformation. The orientation of CovR dimers has been postulated to vary from head-to-head to head-to-tail depending on the promoter [47]. Thus, our discovery that the R144C replacement severely affected CovR function at only particular promoters provides the groundwork for investigating the overall role of the R144 in CovR function as well as further studying different mechanisms by which CovR interacts with diverse promoters.

The finding that strain 10870Δ*covR* and the CovR polymorphic variants had significantly increased transcript levels of genes in the pilus operon compared to wild-type was a surprise given that multiple CovR transcriptome studies have been performed without identifying the pilus genes as being influenced by CovR [22], [32], [36], [48], [49], [50]. These CovR transcriptome studies have mostly been performed in serotype M1 strains except for one study in the serotype M3 strain DSL003 [32]. The nucleotide composition of the pilus promoter region is highly heterogeneous among various GAS strains which may explain the lack of observed CovR influence in previous studies [51]. Given that the pilus regulatory protein Nra is also transcribed from this intergenic region, we cannot say whether CovR directly influences pilus transcription in strain MGAS10870 or is acting indirectly by affecting Nra levels (Figure S1C). A recent study in a serotype M1 strain found that pili diminish GAS virulence in multiple mouse infection models possibly by inducing IL-8 production, neutrophil recruitment, and extracellular DNA-based entrapment [52]. We found that, compared to wild-type, there were increased transcript levels of the gene encoding the IL-8 degrading enzyme SpyCEP and of genes encoding multiple DNase enzymes in the CovR-isoallelic variants (Table S1). Thus, CovR single amino acid replacements in the serotype M3 strain under study here appear to balance augmented immune activation associated with pilus expression with increased immune evasion via IL-8 degradation and degradation of neutrophil extracelluar traps. It was recently reported that *covRS* inactivation in the GAS serotype M49 strain NZ131 resulted in increased pilus production [53]. Bioinformatic analysis of the published genome [54] reveals that strain NZ131 also contains five putative CovR binding sites in the *nra/cpb* intergenic region in this strain, similar to what is observed in strain MGAS10870. Interestingly, elimination of the ATTARA motifs in the *nra*/*cpb* intergenic region only modestly reduced recombinant CovR binding in vitro (Figure 6A) suggesting that CovR binding of DNA is necessary but not always sufficient for gene regulation.

In summary, we have determined the structure of the DNA binding domain of the key streptococcal global regulator CovR and have discovered that single amino acid residue replacements in CovR can affect CovR DNA binding affinity, GAS global gene expression, and strain virulence differentially. These data provide fundamental insights into the mechanisms by which variation in a global regulatory protein influences infection with a major human bacterial pathogen thereby extending understanding into how virulence factor regulation impacts microbial pathogenesis.

## Materials and Methods

### Ethics statement

This study was carried out in strict accordance with the recommendations in the Guide for the Care and Use of Laboratory Animals of the National Institutes of Health. The protocol was approved by Methodist Hospital Research Institute Institutional Animal Care and Use Committee (Protocol Number: AUP-1010-0022). All efforts were made to minimize suffering.

### Bacterial strains and culture media

The strains and plasmids used in this work are presented in Table 3, and primers used for isogenic mutant strain creation are listed in Table S2. Strains were grown in a nutrient-rich medium (Todd-Hewitt broth with 0.2% yeast extract (THY)) at 37 °C with 5% CO_2_. When appropriate, kanamycin and chloramphenicol were added at 200 µg/mL and 10 µg/mL respectively. Strain MGAS10870 is a fully-sequenced, invasive, serotype M3 strain that contains a wild-type *covRS* operon [21]. Strain 10870Δ*covR* was created by replacing the entire CovR DNA binding domain in strain MGAS10870 with a kanamycin resistance cassette via insertional inactivation [22], [55]. Derivatives of strain MGAS10870 that differed only by the presence of a single amino acid replacement were created using the chloramphenicol-resistant, temperature-sensitive plasmid pJL1055 (gift of D. Kasper) as described [30]. For each of the CovR strains derived from strain MGAS10870, sequencing of the entire *covRS* operon, as well as the *mga* and *emm* genes, was performed to check for the presence of spurious mutations that could have developed during the strain construction process (none were found). The *nra*/*cpb* operon of the fully annotated serotype M3 strain MGAS315 [40] is presented (Figure S2A) to facilitate bioinformatic analysis, but strain MGAS315 was not used as the wild-type strain for this study because it contains a single amino replacement in the CovS protein.

### Protein overexpression and purification

CovR and the CovR C-terminal DNA binding domain (CovR_CD_) were amplified from strain MGAS10870 and cloned into pTXB1 as described using primers listed in Table S2 [50]. Recombinant CovR variants were created using site-directed mutagenesis (Stratagene) (primers are listed in Table S2). Overexpression of recombinant forms of the CovR protein was performed in *E. coli* BL21 grown over night at 18°C. Cells derived from three liters of culture were resuspended in 20 mM Tris-HCl pH 8.5, 500 mM NaCl and purified to >95% homogeneity (Figure S4 A) using the IMPACT Protein Purification System (New England Biolabs) which allows for recovery of recombinant CovR isoforms lacking any non-native residues and avoids having to recover proteins from inclusion bodies as part of the purification process [50]. After cleavage and elution with 50 mM DTT, proteins were extensively buffer exchanged into 50 mM CAPS pH 10.0, 100 mM NaCl. CovR_CD_ was further purified by size exclusion chromatography on Superdex 75 in 50 mM CAPS pH 10.0, 50 mM NaCl, 1 mM TCEP (Tris-[2-carboxyethyl] phosphine). Proteins were concentrated to ∼25 mg/ml with Amicon centrifugal filter units (MW cut-off 3kDa) and protein concentrations were assessed by Bradford assay. Selenomethionine-derivatized CovR_CD_ (SeMet-CovR_CD_) was overexpressed using the methionine inhibition pathway and purified as described for native CovR_CD_.

### Crystallization, data collection, and data processing

Crystallization of native and SeMet-CovR_CD_ was carried out at room temperature using the hanging drop vapor-diffusion method with reservoir solutions containing 10–14% PEG 3350 and 50 mM Zinc(OAc)_2_. Native crystals were smaller and did not diffract beyond 2 Å resolution while SeMet crystals diffracted to beyond 1.50 Å. Crystals were cryo-protected using 30% PEG400 and flash frozen in liquid nitrogen. Multiple wavelength anomalous dispersion (MAD) diffraction data using SeMet-CovR_CD_ crystals were collected under cryogenic conditions at the Advanced Light Source (ALS), Berkeley, Beamline 8.3.1. Data were processed with MOSFLM [56] and Scala in the CCP4 suite (Table 2). Crystals take the space group P2_1_ with cell dimensions of a = 30.5 Å, b = 36.7 Å, c = 38.9 Å and α = 90.0°, β = 94.6°, γ = 90.0°.

### Structure determination and refinement

The CovR_CD_ structure was determined to 1.5 Å resolution by MAD phasing [27]. Four selenium sites were located using the program SOLVE [57] and solvent flattening and initial model building were performed using RESOLVE [58]. Given the isomorphism of the SeMet-CovR_CD_ and native CovR_CD_ crystals and the significantly higher resolution of the former protein, we refined only the SeMet-CovR_CD_ structure. The asymmetric unit contains one CovR_CD_ molecule. The structure was refined to final R_work_ and R_free_ values of 21.60% and 23.64%, respectively, by multiple rounds of model building in COOT [59] and positional and B-factor refinement with CNS 1.2 [60]. The resulting model showed excellent geometry (Table 2) and contains residues 134-228 including the initial methionine as well as 107 water molecules. Selected data collection, phasing and refinement statistics are given in Table 2. Crystallization-related figures were created using Pymol [61].

### RNA isolation, TaqMan transcript level analysis, and expression microarray analysis

RNA was purified using an RNeasy mini kit (Qiagen). A custom-made Affymetrix GeneChip that contains 100% of the ORFs of strain MGAS10870 was used for expression microarray (transcriptome) studies as described [62]. To compare gene transcript levels between the wild-type and the various derivative strains, a two-sample *t*-test (unequal variance) was applied with a *P* value of <0.05 and a mean transcript level of at least 1.5-fold being considered statistically significant. TaqMan real-time QRT-PCR (primers and probes listed Table S2) was performed on an Applied Biosystems 7500 system as previously described [63]. All QRT-PCR samples were performed in duplicate on two separate occasions and analyzed in duplicate.

### CovR phosphorylation and detection of phosphorylated proteins

Wt and mutant CovR proteins were phosphorylated with acetyl phosphate as described [17]. The phosphorylation state was verified using Phos-tag Acrylamide gels (WAKO chemicals, USA, Inc.).

### Electrophoretic mobility shift assays

∼300 bps of the promoter regions of selected CovR regulated genes were amplified by PCR from indicated GAS strains (primers are listed Table S2). A variant of the *nra/cpb* intergenic region in which all five putative CovR binding sites are mutated (A**TT**AR**A** -> A**GG**AR**C**) was synthesized and cloned into pUC57 by Genscript USA Inc. and PCR amplified as described. The resultant PCR products (0.25 µg/ml) were incubated with increasing amounts of phosphorylated wild-type CovR (0.25–1 µM) and CovR variant proteins (0.25–5 µM) at 37°C for 15 min in TBE buffer containing 89 mM Tris, 89 mM borate, 1 mM EDTA, and 5% glycerol and 10 µg/ml polydI:dC to account for non-specific DNA binding. Samples were then separated by native gel electrophoresis on a 6% TBE gel for 70 min at 110 V and stained with ethidium bromide.

### Circular dichroism spectroscopy

Wild type and mutant CovR proteins were diluted to a concentration of 0.4 mg/ml in 10 mM potassium phosphate, pH 7.5, 10 mM NaF prior to measurements. Far-UV (200–260 nm) spectra were recorded on a Jasco J-810 spectropolarimeter at 37°C and a scan speed of 20 nm/min. Three scans were averaged and the averaged blank spectra were substracted.

### Mouse infection studies

Mouse experiments were performed according to protocols approved by the Methodist Hospital Research Institute Institutional Animal Care and Use Committee. 20 female outbred CD-1 Swiss mice (Harlan-Sprague-Dawley) were injected intraperitoneally with 1.0×10^7^ GAS CFU and monitored for near-mortality.

### Data deposition footnote

Expression microarray data have been deposited at Gene Expression Omnibus (GEO) database at NCBI http://www.ncbi.nlm.nih.gov/geo/accession number GSE32106.

Coordinates and structure factors are deposited in the Protein Data Bank under PDB ID code 3RJP.

### Gene IDs [Entrez-Gene numbers]

*covR*, 3572611; *covS*, 3572612; *slo*, 3572764; *spyCEP*, 3760194; *sagA*, 3572347; *hasA*, 3571023; *ska*, 3571199; *speC*, 900896; *nra*, 1008411; *cbp*, 1008412

## Supporting Information

Figure S1 **Additional QRT-PCR data.** For all panels indicated strains were grown as described in THY, and RNA was harvested, converted to cDNA and analyzed for indicated gene transcript level via TaqMan QRT-PCR. In each panel, there are three bars for each strain corresponding from left-to-right to mid-exponential, late-exponential, and stationary growth phase respectively (see Fig. 3 A). Data graphed are mean ± standard deviation of duplicate biologic replicates performed on two separate occasions and analyzed in duplicate (total of 8 data points) of transcript level in the indicated strain relative to the wild-type strain MGAS10870. (A) *spyCEP* (gene encoding IL-8 degrading enzyme), (B) spyM3_0099 (gene in pilus operon), and (C) *nra* (gene encoding pilus regulatory protein).
(TIF)

Figure S2 **Depiction of pilus operon and** ***nra/cpb*** **intergenic region in the serotype M3 strain MGAS10870.** The structure of the pilus operon including the gene encoding the transcriptional regulator Nra is shown at top. Numbers correspond to gene designation in the serotype M3 strain MGAS315. Details of the *nra/cpb* intergenic region are shown in bottom aspect including the Nra start codon (red), putative CovR binding sites (blue), and the Cpb start codon (purple). Asterisks indicate the location of an A/T deletion in the serotype M3 strain MGAS315 compared to strain MGAS10870. This deletion is not present in the fully sequenced serotype M3 strain SS-I (i.e. strain SS-I has an *nra/cpb* intergenic sequence identical to that of strain MGAS10870).
(TIF)

Figure S3 **Electrophoretic mobility-shift assay (EMSA) of recombinant CovR DNA binding.** (A) DNA from the *cpb/nra* promoter region of indicated strains was incubated with increasing amounts of recombinant, phosphorylated wild-type CovR (amount shown in µM). (B) DNA from the *ska* promoter region of strain MGAS10870 was incubated with increasing amounts of phosphorylated CovR wild type, CovR-R144C, and CovR-R158C (amount shown in µM). For (A and B) the samples were incubated at 37°C for 15 min and electrophoresed at 110 V for 70 min. The resulting 6% polyacrylamide gel was stained with ethidium bromide. ns, nonspecific DNA; f, non-complexed/free DNA; cI, lower molecular weight complex; cII higher molecular weight complex. Gel shown is representative of identical results obtained on three separate occasions.
(TIF)

Figure S4 **Analysis of recombinant CovR variants.** (A) SDS-PAGE analysis of full-length, recombinant CovR variants. From left to right; wild-type CovR, CovR with R144C single amino acid replacement, and CovR with R158C single amino acid replacement. (B) Analysis of secondary structure of recombinant CovR proteins. Circular dichroism spectroscopy (CD). Far-UV (200–260 nm) CD spectra of CovR wild type (red), CovR-R144C (blue), and CovR-R158 (green).
(TIF)

Table S1 **Summary of gene transcript levels in indicated strains compared to the parental, wild-type strain MGAS10870.**
(DOC)

Table S2 **Primers and probes used in this study.**
(DOC)
