# Supplementary figures and images for: Distinct Single Amino Acid Replacements in the Control of Virulence Regulator Protein Differentially Impact Streptococcal Pathogenesis
Source: PLoS Pathog. 2011 Oct 20;7(10):e1002311. doi: 10.1371/journal.ppat.1002311 (PMC3197619; doi:10.1371/journal.ppat.1002311)

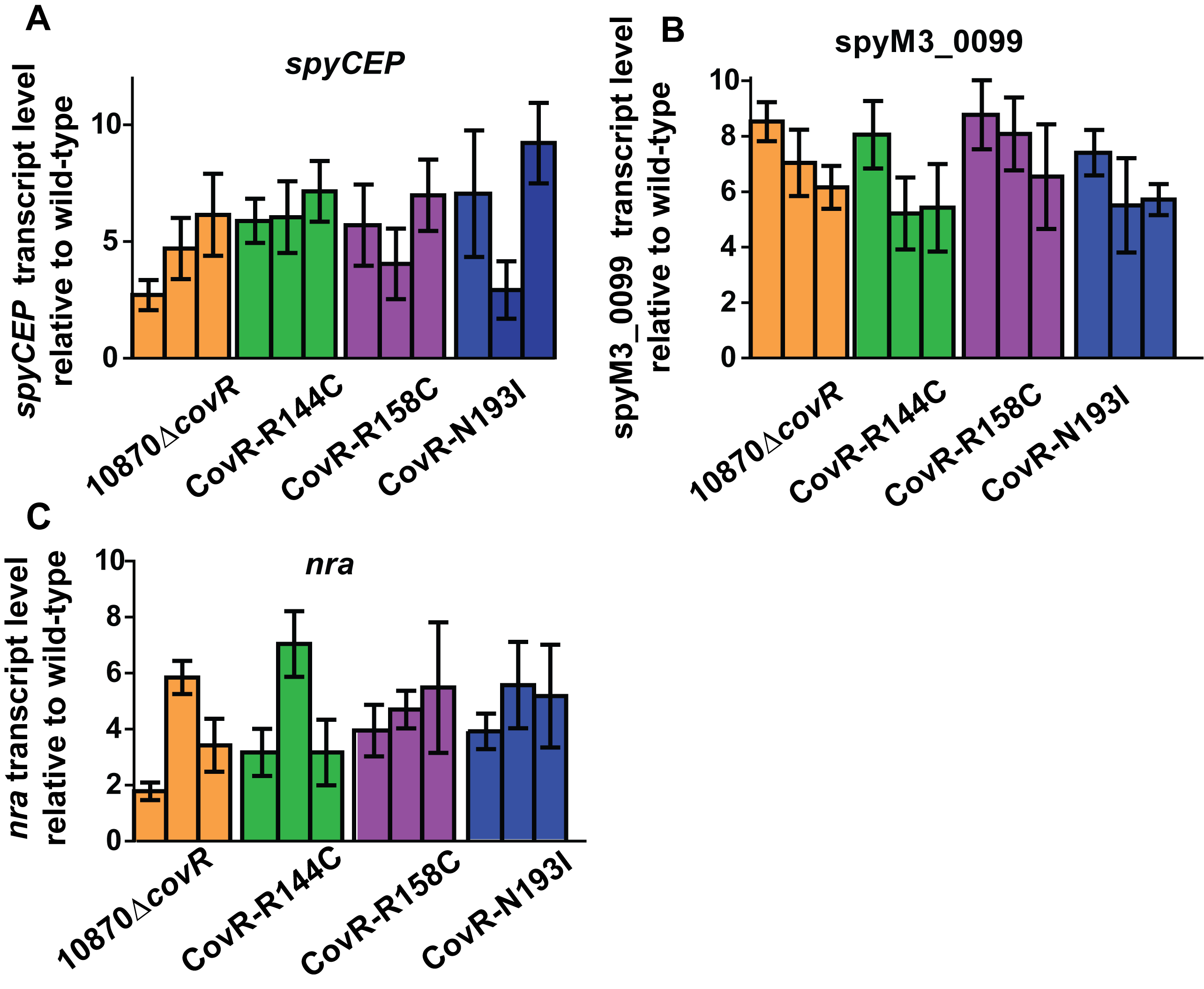

Supplement: Figure S1 — Additional QRT-PCR data. For all panels indicated strains were grown as described in THY, and RNA was harvested, converted to cDNA and analyzed for indicated gene transcript level via TaqMan QRT-PCR. In each panel, there are three bars for each strain corresponding from left-to-right to mid-exponential, late-exponential, and stationary growth phase respectively (see Fig. 3 A). Data graphed are mean ± standard deviation of duplicate biologic replicates performed on two separate occasions and analyzed in duplicate (total of 8 data points) of transcript level in the indicated strain relative to the wild-type strain MGAS10870. (A) spyCEP (gene encoding IL-8 degrading enzyme), (B) spyM3_0099 (gene in pilus operon), and (C) nra (gene encoding pilus regulatory protein). (TIF) [file ppat.1002311.s001.tif]

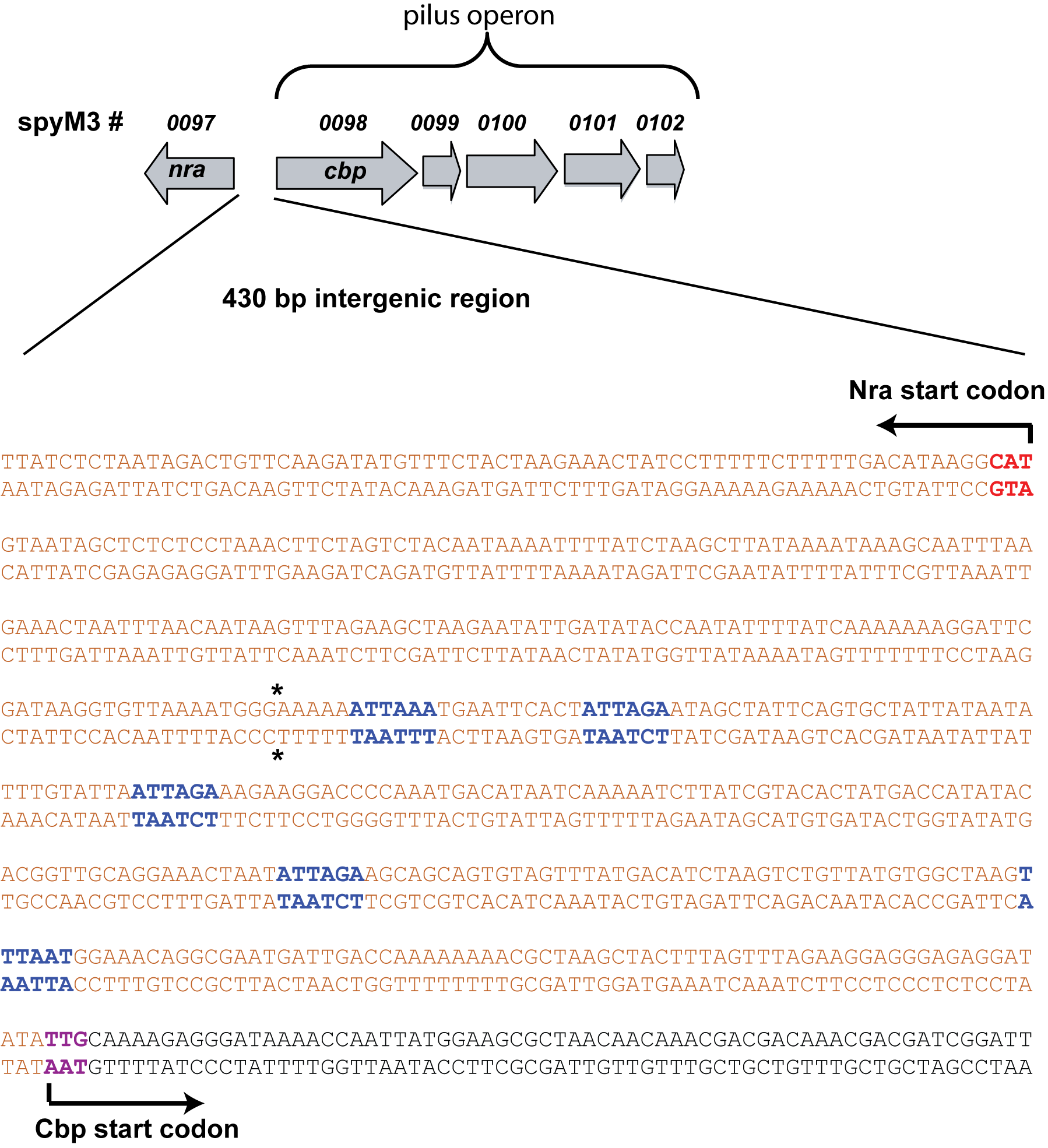

Supplement: Figure S2 — Depiction of pilus operon and nra/cpb intergenic region in the serotype M3 strain MGAS10870. The structure of the pilus operon including the gene encoding the transcriptional regulator Nra is shown at top. Numbers correspond to gene designation in the serotype M3 strain MGAS315. Details of the nra/cpb intergenic region are shown in bottom aspect including the Nra start codon (red), putative CovR binding sites (blue), and the Cpb start codon (purple). Asterisks indicate the location of an A/T deletion in the serotype M3 strain MGAS315 compared to strain MGAS10870. This deletion is not present in the fully sequenced serotype M3 strain SS-I (i.e. strain SS-I has an nra/cpb intergenic sequence identical to that of strain MGAS10870). (TIF) [file ppat.1002311.s002.tif]

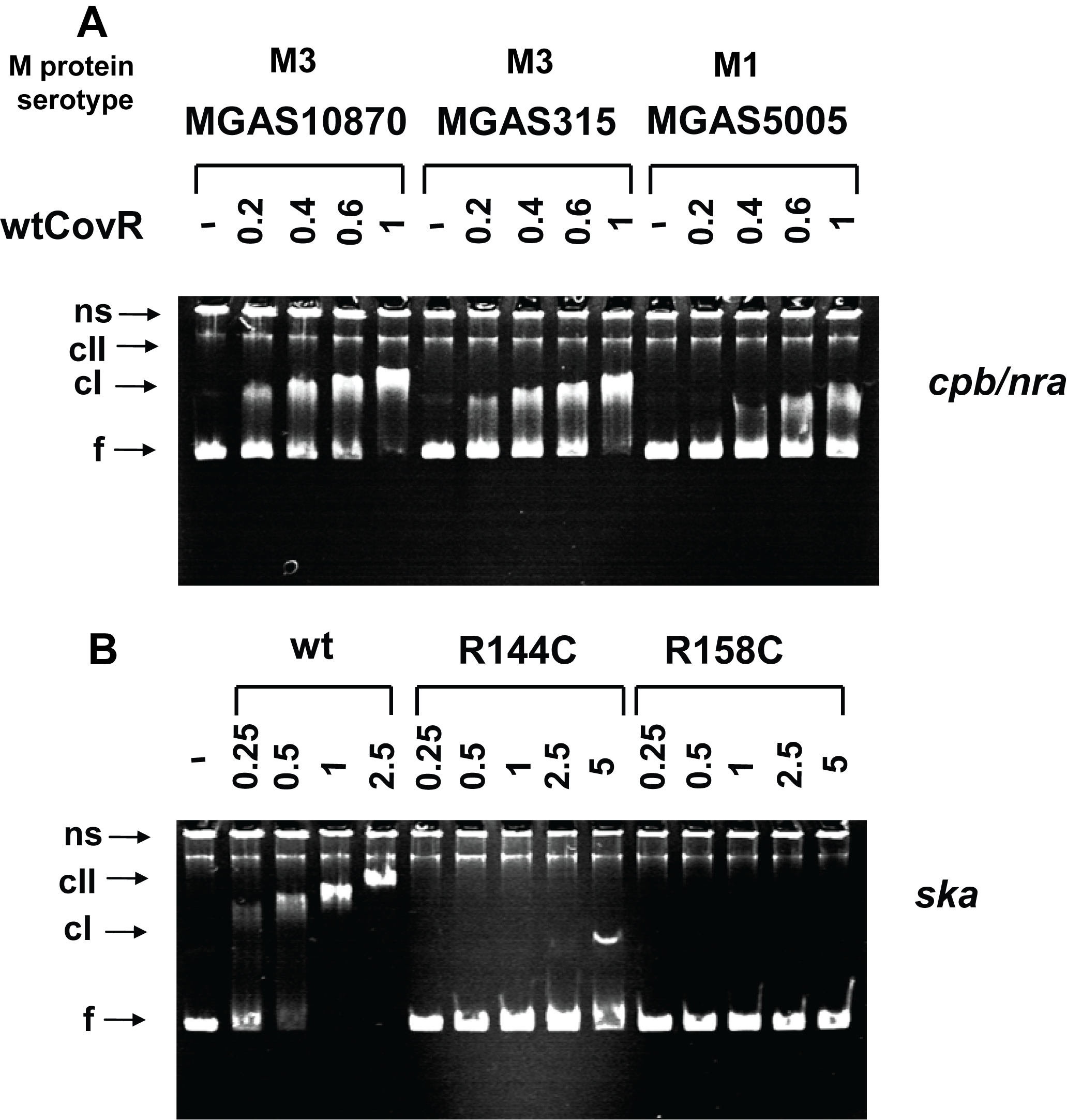

Supplement: Figure S3 — Electrophoretic mobility-shift assay (EMSA) of recombinant CovR DNA binding. (A) DNA from the cpb/nra promoter region of indicated strains was incubated with increasing amounts of recombinant, phosphorylated wild-type CovR (amount shown in µM). (B) DNA from the ska promoter region of strain MGAS10870 was incubated with increasing amounts of phosphorylated CovR wild type, CovR-R144C, and CovR-R158C (amount shown in µM). For (A and B) the samples were incubated at 37°C for 15 min and electrophoresed at 110 V for 70 min. The resulting 6% polyacrylamide gel was stained with ethidium bromide. ns, nonspecific DNA; f, non-complexed/free DNA; cI, lower molecular weight complex; cII higher molecular weight complex. Gel shown is representative of identical results obtained on three separate occasions. (TIF) [file ppat.1002311.s003.tif]

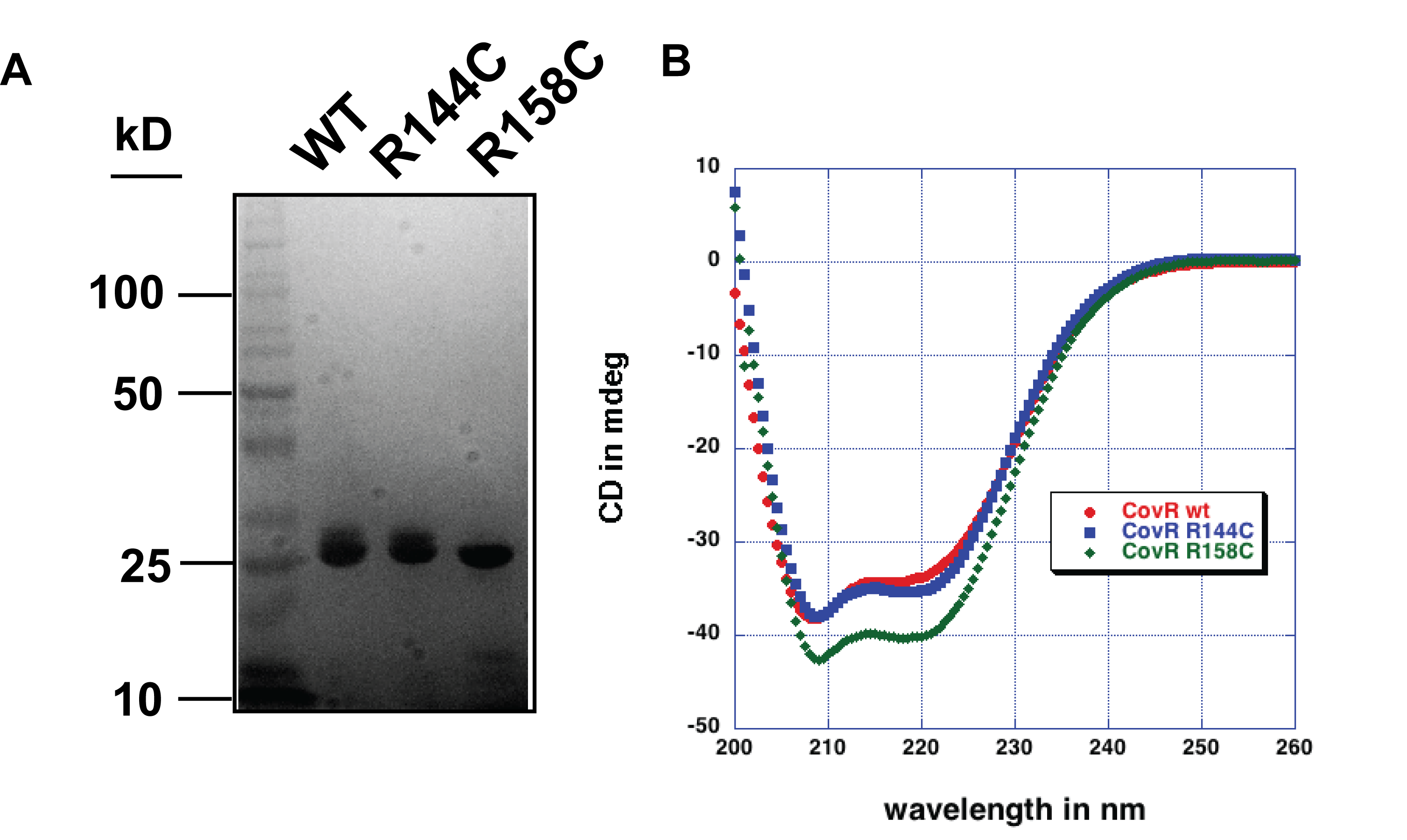

Supplement: Figure S4 — Analysis of recombinant CovR variants. (A) SDS-PAGE analysis of full-length, recombinant CovR variants. From left to right; wild-type CovR, CovR with R144C single amino acid replacement, and CovR with R158C single amino acid replacement. (B) Analysis of secondary structure of recombinant CovR proteins. Circular dichroism spectroscopy (CD). Far-UV (200–260 nm) CD spectra of CovR wild type (red), CovR-R144C (blue), and CovR-R158 (green). (TIF) [file ppat.1002311.s004.tif]
