# Supplementary material for: Distinct Single Amino Acid Replacements in the Control of Virulence Regulator Protein Differentially Impact Streptococcal Pathogenesis
Source: PLoS Pathog. 2011 Oct 20;7(10):e1002311. doi: 10.1371/journal.ppat.1002311 (PMC3197619; doi:10.1371/journal.ppat.1002311)
Supplement: Table S1 — Summary of gene transcript levels in indicated strains compared to the parental, wild-type strain MGAS10870. (DOC) [file ppat.1002311.s005.doc]

**Table S1**. Summary of gene transcript levels in indicated strains compared to the parental, wild-type strain MGAS10870.

| **Gene number** | **Gene name** | **Protein function** | **10870 Δ*covR*** | **CovR-R144C** | **CovR-R158C** | **CovR-N193I** |
| --- | --- | --- | --- | --- | --- | --- |
| SpyM3_0023 | *purN* | Phosphoribosylglycinamide formyltransferase | -1.55^a^ |  |  |  |
| SpyM3_0027 | *purE* | Phosphoribosylaminoimidazole carboxylase | -1.75 |  |  |  |
| SpyM3_0031 | *ruvB* | Transcriptional regulator | -1.65 |  |  |  |
| SpyM3_0036 |  | Alcohol dehydrogenase |  | +1.5 | +1.50 | +1.63 |
| SpyM3_0097 | *nra* | Transcriptional regulator |  | +1.87 | +1.80 |  |
| SpyM3_0098 | *cpa* | Fibronectin binding protein | +12.26 | +8.21 | 12.62 | +12.69 |
| SpyM3_0099 | *sipA2* | Signal peptidase I | +4.54 | +5.22 | +4.36 | +5.00 |
| SpyM3_0100 | *tee3* | Fibronectin binding protein | +5.62 | +5.46 | +4.82 | +5.72 |
| SpyM3_0101 | *srtC2* | Sortase B familiy protein | +7.87 | +7.17 | +6.30 | +6.84 |
| SpyM3_0102 | *orfB* | Hypothetical protein | +5.43 | +5.10 | +4..73 | +5.23 |
| SpyM3_0105 |  | Hypothetical protein | +5.03 | +4.03 | +4.15 | +4.92 |
| SpyM3_0131 |  | Hypothetical protein | +3.00 | +2.53 | +3.17 | +3.13 |
| SpyM3_0133 | *metB* | Cystathionine beta-lyase | +8.22 | +3.66 | +8.28 | +8.8 |
| SpyM3_0150 |  | Zinc finger protein | -1.73 |  |  |  |
| SpyM3_0151 |  | BioY protein | -1.71 |  |  | -2.07 |
| SpyM3_0162 |  | MarR regulator | +3.65 | +2.57 | +3.30 | +2.24 |
| SpyM3_0163 |  | Multi-drug resistance transporter | +1.96 | +1.67 | +1.95 |  |
| SpyM3_0164 |  | Multi-drug resistance transporter | +3.01 | +2.07 | +2.79 | +1.79 |
| SpyM3_0169 |  | Hypothetical protein | +1.65 |  | +1.62 |  |
| SpyM3_0237 |  | Serine threonine symporter | -1.79 |  |  |  |
| SpyM3_0243 |  | Hypothetical protein | +1.83 |  | +1.59 | +1.52 |
| SpyM3_0245 | *covS* | Histidine kinase | +2.2 |  |  | +1.67 |
| SpyM3_0246 |  | Regulatory protein | +1.68 |  |  |  |
| SpyM3_0247 | *dnaB* | DNA helicase | +1.54 |  |  |  |
| SpyM3_0299 |  | Permease | -1.53 | +1.58 |  |  |
| SpyM3_0301 | *nrdF.1* | Ribonucleoside-diphosphate reductase |  | -1.73 |  |  |
| SpyM3_0303 | *nrdE.1* | Ribonucleoside-diphosphate reductase |  | -1.6 |  |  |
| SpyM3_0305 |  | Hypothetical membrane associated protein |  | +1.60 |  |  |
| SpyM3_0311 |  | Phosphoglycerate transporter protein |  |  | +1.68 |  |
| SpyM3_0402 |  | Multidrug resistance protein B | +4.63 |  |  |  |
| SpyM3_0403 | *licT* | Transcription antiterminator, BglG family |  | +2.42 |  |  |
| SpyM3_0422 |  | Phosphoglycerate mutase family protein | +1.53 |  |  | +1.56 |
| SpyM3_0423 |  | Transcriptional regulator |  |  |  | +1.51 |
| SpyM3_0425 |  | N-acetylmuramoyl-L-alanine amidase | +1.73 |  | +1.78 |  |
| SpyM3_0453 |  | Iron-sulfur cluster-binding protein | +1.84 |  | +1.87 | +1.94 |
| SpyM3_0466 | *adcA* | High-affinity zinc uptake system protein | +1.72 |  |  |  |
| SpyM3_0467 |  | Transcriptional regulator, GntR family | +2.63 |  | +2.04 | +2.50 |
| SpyM3_0468 | *agaS* | Galactosamine-6-phosphate deaminase | +2.08 |  | +1.80 | +2.71 |
| SpyM3_0480 | *sagA* | Streptolysin S precursor | +3.18 | +2.64 | +2.66 | +3.30 |
| SpyM3_0481 | *sagB* | Streptolysin S biosynthesis protein SagB | +2.96 | +2.93 | +3.97 | +3.73 |
| SpyM3_0482 | *sagC* | Streptolysin S biosynthesis protein SagC | +2.72 | +2.88 | +3.94 | +3.45 |
| SpyM3_0483 | *sagD* | Streptolysin S biosynthesis protein SagD | +2.38 | +2.58 | +3.03 | +2.99 |
| SpyM3_0484 | *sagE* | Streptolysin S putative self-immunity protein | +3.16 |  | +3.73 | +3.80 |
| SpyM3_0485 | *sagF* | Streptolysin S biosynthesis protein | +1.90 |  | +2.48 | +2.29 |
| SpyM3_0486 | *sagG* | Streptolysin S export ATP-binding protein | +2.61 | +2.50 | +3.18 | +3.35 |
| SpyM3_0487 | *sagH* | Streptolysin S export transmembrane protein | +2.36 | +2.43 | +3.11 | +2.87 |
| SpyM3_0488 | *sagI* | Streptolysin S export transmembrane protein | +2.72 | +2.55 | +3.09 | +2.99 |
| SpyM3_0535 |  | Ferredoxin | -1.71 |  |  |  |
| SpyM3_0569 |  | Cell surface protein | -2.90 | -1.55 | -2.27 | -3.18 |
| SpyM3_0570 |  | Hypothetical cytosolic protein | -2.99 | -1.50 | -2.13 | -3.04 |
| SpyM3_0580 | *fruA* | PTS system, fructose-specific IIABC | -2.42 |  |  |  |
| SpyM3_0582 |  | Hypothetical protein | +1.59 |  | +1.54 | +1.55 |
| SpyM3_0589 |  | Hypothetical protein |  |  |  | -3.79 |
| SpyM3_0590 | *fms* | Peptide deformylase |  |  |  | -3.13 |
| SpyM3_0591 |  | 5 –nucleotidase | -1.98 |  |  | -2.33 |
| SpyM3_0629 |  | Hypothetical protein | +1.55 |  |  |  |
| SpyM3_0630 |  | Rhodanese-related sulfurtransferases | +1.68 |  |  |  |
| SpyM3_0653 |  | ABC transporter substrate-binding protein | -1.58 |  |  |  |
| SpyM3_0654 |  | Hypothetical protein | -2.22 |  |  | -2.24 |
| SpyM3_0655 |  | ABC transporter permease protein | -1.72 |  |  |  |
| SpyM3_0725 |  | Hyaluronoglucosaminidase | -1.97 | +1.67 |  |  |
| SpyM3_0726 |  | Phage infection protein | -3.17 | +2.33 |  | -2.72 |
| SpyM3_0727 |  | Phage protein | -4.16 | +2.00 |  | -3.34 |
| SpyM3_0730 |  | Holin | -1.97 | +1.67 |  | -2.02 |
| SpyM3_0741 |  | PTS system, mannose fructose family IIA | +3.30 | +3.30 | +5.17 | +5.21 |
| SpyM3_0742 | *ptsB* | PTS system, mannose fructose family IIB | +2.62 | +2.20 | +3.37 | +3.49 |
| SpyM3_0743 | *ptsC* | PTS system, mannose fructose family IIC | +3.19 | +2.78 | +4.45 | +4.31 |
| SpyM3_0744 | *ptsD* | PTS system, mannose fructose family IID | +2.49 | +2.38 | +3.34 | +3.50 |
| SpyM3_0746 | *yesN* | Response regulator |  |  | +1.74 | +1.53 |
| SpyM3_0788 |  | Short chain dehydrogenase |  |  | +1.70 | +1.64 |
| SpyM3_0789 |  | Short chain dehydrogenase |  |  | +1.50 |  |
| SpyM3_0794 | *xpt* | Xanthine phosphoribosyltransferase | -1.52 |  |  | -2.13 |
| SpyM3_0796 |  | Iron-sulfur cluster assembly repair protein | -1.61 |  |  |  |
| SpyM3_0848 |  | Hypothetical membrane spanning protein | -1.55 |  |  |  |
| SpyM3_0849 | *pdxK* | Pyridoxine kinase | -1.60 |  |  |  |
| SpyM3_0858 |  | Luciferase-like monooxygenase |  | +1.57 |  |  |
| SpyM3_0859 |  | Probable NADH-dependent flavin oxidoreductase | +1.81 |  | +1.66 |  |
| SpyM3_0871 | *coaA* | Pantothenate kinase |  |  | +1.73 |  |
| SpyM3_0879 | *pstA* | Phosphate transport system permease | +1.51 |  | +1.52 |  |
| SpyM3_0880 | *pstC* | Phosphate transport system permease | +1.53 |  |  |  |
| SpyM3_0881 | *pstS* | Phosphate-binding protein |  |  | +1.53 |  |
| SpyM3_0903 |  | Na(+)-linked D-alanine glycine permease |  | +1.58 |  |  |
| SpyM3_0923 |  | Holin | -1.79 | +1.83 |  |  |
| SpyM3_0927 |  | Phage infection protein | -13.84 |  | -6.53 | -10.56 |
| SpyM3_0928 |  | Hyaluronoglucosaminidase | -3.54 |  | -2.68 | -3.889 |
| SpyM3_0929 |  | Hyaluronoglucosaminidase |  |  | -2.30 | -2.113 |
| SpyM3_0950 |  | Phage encoded transcriptional regulator | -2.72 |  | -2.76 | -3.20 |
| SpyM3_0960 |  | Phage protein | -2.43 |  | -2.05 | -3.19 |
| SpyM3_0963 |  | Phage protein |  |  |  | -2.07 |
| SpyM3_0965 |  | Phage protein | -1.71 |  |  |  |
| SpyM3_1008 |  | Nicotinamide mononucleotide transporter | -1.78 |  |  | -2.00 |
| SpyM3_1013 |  | Nicotinate-nucleotide pyrophosphorylase | -1.59 |  |  | +1.59 |
| SpyM3_1032 | *grab* | Protein G-related alpha 2M-binding protein | +1.87 |  | +1.65 | +1.95 |
| SpyM3_1040 |  | Hypothetical membrane spanning protein | +2.62 | +2.00 | +2.33 | +2.54 |
| SpyM3_1055 |  | CAAX amino terminal protease family | -1.60 |  |  |  |
| SpyM3_1065 | *nagB* | Glucosamine-6-phosphate isomerase |  | +1.51 | +4.75 | -4.36 |
| SpyM3_1068 |  | Peptidase family S11 | +14.71 |  |  |  |
| SpyM3_1078 |  | Kup system potassium uptake protein | +11.63 |  | +2.28 | +2.79 |
| SpyM3_1094 |  | Phage protein | +1.53 |  | +1.55 | -1.64 |
| SpyM3_1095 | *mf4.1* | Deoxyribonuclease precursor | +2.16 | +1.70 | +1.90 | +1.88 |
| SpyM3_1096 |  | Phage protein |  | -1.61 |  |  |
| SpyM3_1161 |  | Arginine transport system permease | -1.68 |  |  |  |
| SpyM3_1187 |  | ATP-dependent endopeptidase Lon | +1.51 |  |  |  |
| SpyM3_1188 |  | Phosphopantetheine adenylyltransferase | +1.62 |  |  |  |
| SpyM3_1189 |  | Methyltransferase | +1.60 |  |  |  |
| SpyM3_1190 | *asnA* | Aspartate--ammonia ligase | +4.03 |  | +2.41 | +2.38 |
| SpyM3_1191 | *arcC* | Carbamate kinase |  | +4.65 | +2.58 | +4.91 |
| SpyM3_1192 |  | Xaa-His dipeptidase |  |  | +1.55 | +2.77 |
| SpyM3_1193 |  | Arginine ornithine antiporter |  | +2.85 |  | +2.98 |
| SpyM3_1194 | *arcB* | Ornithine carbamoyltransferase |  |  |  | +2.46 |
| SpyM3_1195 |  | Acetyltransferase |  | +3.62 |  | +3.52 |
| SpyM3_1196 | *arcA* | Arginine deiminase |  | +4.19 | +1.80 | +4.09 |
| SpyM3_1201 | *zmpS* | Histidine kinase |  |  | +1.53 |  |
| SpyM3_1203 |  | Phage protein | +1.63 | +1.67 | +1.93 | +1.50 |
| SpyM3_1204 | *slaA* | Phospholipase | +1.63 | +1.70 | +1.71 | +1.56 |
| SpyM3_1208 |  | Phage-associated cell wall hydrolase | -2.39 |  |  | -2.14 |
| SpyM3_1209 |  | Phage protein | -1.96 |  | -2.02 | -2.36 |
| SpyM3_1210 |  | Phage protein | -1.82 |  |  | -2.09 |
| SpyM3_1211 |  | Phage protein | -2.87 |  |  | -2.76 |
| SpyM3_1215 |  | Phage endopeptidase | -10.90 |  | -4.23 | -13.30 |
| SpyM3_1216 |  | Phage protein | -3.74 |  | -3.63 | -18.08 |
| SpyM3_1217 |  | Phage protein | -3.12 |  | -2.77 | -3.41 |
| SpyM3_1219 |  | Phage protein | -2.71 |  | -2.53 | -3.86 |
| SpyM3_1220 |  | Major tail shaft protein |  |  | -2.23 |  |
| SpyM3_1221 |  | Minor capsid protein | -5.09 |  | -2.71 | -4.31 |
| SpyM3_1222 |  | Minor capsid protein | -3.57 |  | -2.80 | -3.65 |
| SpyM3_1224 |  | Phage protein | -3.27 |  | -2.25 | -3.85 |
| SpyM3_1226 |  | Phage protein | -4.18 |  | -2.71 | -4.69 |
| SpyM3_1228 |  | Phage protein | -1.75 |  |  |  |
| SpyM3_1230 |  | Minor capsid protein |  |  | -2.20 | -2.50 |
| SpyM3_1231 |  | Terminase large subunit | -1.90 |  | -2.11 | -2.43 |
| SpyM3_1233 |  | Phage transcriptional regulator | -1.80 |  |  |  |
| SpyM3_1234 |  | Phage protein | -2.16 |  | -2.77 | -2.84 |
| SpyM3_1235 |  | ABC transporter ATP-binding protein | -2.88 |  | -2.50 |  |
| SpyM3_1236 |  | Chromosome partitioning protein parB | -3.95 |  | -2.79 | -6.90 |
| SpyM3_1238 |  | Phage protein | -0.64 |  |  | +1.73 |
| SpyM3_1239 |  | Phage protein | -2.43 |  | -2.35 | -2.73 |
| SpyM3_1241 |  | Phage protein | -2.28 |  | -2.31 | -2.62 |
| SpyM3_1243 |  | Phage protein | -1.69 |  |  | -2.12 |
| SpyM3_1244 |  | Phage protein | -2.54 |  | -2.40 | -3.46 |
| SpyM3_1246 |  | Phage protein | -2.05 |  | -2.09 | -2.22 |
| SpyM3_1249 |  | Phage single-strand DNA binding protein | -2.35 |  | -2.15 | -2.80 |
| SpyM3_1250 |  | Phage single-strand DNA binding protein | -1.91 |  | -2.25 | -2.07 |
| SpyM3_1251 |  | Phage protein | -3.06 |  | -2.94 | -3.55 |
| SpyM3_1252 |  | Phage protein | -4.09 |  | -4.59 | -18.75 |
| SpyM3_1253 |  | Phage protein | -3.22 |  |  | -5.24 |
| SpyM3_1254 |  | DNA replication protein dnaD | -1.62 |  | -2.13 | -2.46 |
| SpyM3_1255 |  | Phage replication protein | -2.26 |  | -2.94 | -2.90 |
| SpyM3_1256 |  | Phage protein | -2.79 |  | -4.33 | -4.94 |
| SpyM3_1257 |  | Phage protein | -2.79 |  | -2.07 | -4.33 |
| SpyM3_1258 |  | Phage protein | -2.32 |  | -3.41 | -3.22 |
| SpyM3_1260 |  | Phage protein | -2.02 |  | -2.06 | -2.16 |
| SpyM3_1261 |  | Phage antirepressor protein | -2.76 |  | -2.64 | -4.01 |
| SpyM3_1262 |  | Phage protein | -4.01 |  | -2.71 | -4.30 |
| SpyM3_1285 | *lacZ* | Beta-galactosidase |  |  | +1.57 |  |
| SpyM3_1286 | *trxR* | Response regulator |  |  | +1.81 |  |
| SpyM3_1287 | *trxS* | Histidine kinase |  |  | +1.77 |  |
| SpyM3_1288 |  | Hypothetical membrane spanning protein | +2.01 |  |  | +2.21 |
| SpyM3_1301 | *speA* | Enterotoxin (speA) | +1.73 | +1.76 | +1.88 | +1.99 |
| SpyM3_1330 |  | Phage encoded transcriptional regulator | -1.66 |  |  |  |
| SpyM3_1331 |  | Phage protein | -2.32 |  |  |  |
| SpyM3_1332 |  | Phage protein | -1.85 |  |  |  |
| SpyM3_1336 |  | Phage protein | -2.31 |  | -2.05 | -2.25 |
| SpyM3_1339 |  | DNA primase | -2.49 |  | -2.80 |  |
| SpyM3_1348 |  | Phage protein | -1.75 |  |  | -2.56 |
| SpyM3_1351 |  | Phage protein |  |  | +1.53 | +1.55 |
| SpyM3_1352 |  | Phage protein | +1.77 | +1.62 | +2.00 | +1.87 |
| SpyM3_1353 |  | Phage protein | +2.02 | +1.76 | +2.27 | +2.10 |
| SpyM3_1354 |  | DNA integration recombination protein | +2.18 | +1.6 | +2.04 | +2.08 |
| SpyM3_1355 |  | Regulatory protein recX | +2.02 |  | +1.89 | +1.88 |
| SpyM3_1357 |  | Hypothetical protein |  |  | -4.03 |  |
| SpyM3_1409 | *sdn* | Streptodornase |  |  | +1.77 | +1.60 |
| SpyM3_1413 |  | Holin | -2.20 | +1.80 |  |  |
| SpyM3_1415 |  | Phage protein | -1.91 |  |  |  |
| SpyM3_1417 |  | Phage infection protein | -2.31 | +2.01 |  | -2.53 |
| SpyM3_1419 |  | Phage endopeptidase | -2.38 | +2.10 |  | -2.55 |
| SpyM3_1420 |  | Phage protein | -1.86 |  |  | -2.04 |
| SpyM3_1421 |  | Phage protein | -2.33 | +1.77 |  |  |
| SpyM3_1423 |  | Phage protein | -2.30 | +1.78 |  | -2.28 |
| SpyM3_1424 |  | Phage protein |  | +1.88 |  |  |
| SpyM3_1425 |  | Phage protein | -3.47 | +1.94 | -2.16 | -3.39 |
| SpyM3_1426 |  | Phage protein | -2.03 | +1.73 |  | -2.00 |
| SpyM3_1427 |  | Phage protein | -3.52 | +2.10 |  | -3.10 |
| SpyM3_1428 |  | Phage protein | -3.80 | +2.06 | -2.00 | -2.91 |
| SpyM3_1429 |  | Phage protein | -2.18 | +1.86 |  |  |
| SpyM3_1430 |  | Phage scaffold protein | -3.09 | +1.86 |  | -2.95 |
| SpyM3_1432 |  | Phage protein | -2.60 |  | -2.21 | -3.08 |
| SpyM3_1433 |  | Phage protein | -1.91 | +1.77 |  | -2.38 |
| SpyM3_1434 |  | Portal protein | -2.65 |  | -2.19 | -3.27 |
| SpyM3_1435 |  | Terminase large subunit | -3.97 |  | -3.35 | -6.33 |
| SpyM3_1436 |  | Phage Terminase Small Subunit | -3.08 |  |  | -2.68 |
| SpyM3_1437 |  | Phage transcriptional activator | -2.54 |  | -2.79 | -2.99 |
| SpyM3_1438 |  | Phage protein | -3.09 |  | -2.74 | -4.33 |
| SpyM3_1439 |  | Phage protein |  |  |  | -7.27 |
| SpyM3_1440 |  | Phage-related DNA helicase | -2.61 |  | -2.54 | -13.57 |
| SpyM3_1442 |  | DNA primase | -2.06 |  | -2.14 | -2.35 |
| SpyM3_1443 |  | Phage-related DNA polymerase | -2.55 |  | -2.25 | -3.70 |
| SpyM3_1446 |  | Phage protein |  |  | -2.06 |  |
| SpyM3_1447 |  | Phage protein |  |  | -2.71 | -2.15 |
| SpyM3_1448 |  | Phage protein | -2.12 |  | -2.42 | -3.59 |
| SpyM3_1450 |  | Phage protein | -1.66 |  |  |  |
| SpyM3_1452 |  | Phage protein |  |  |  | -2.01 |
| SpyM3_1453 |  | Phage protein | -2.24 |  | -2.35 | -3.90 |
| SpyM3_1454 |  | Phage protein | -2.14 |  | -2.00 | -2.63 |
| SpyM3_1488 |  | PTS system, galactose-specific IIA component | |  | +1.55 |  |
| SpyM3_1493 |  | Esterase (EC 3.1.1.-) | -2.68 |  |  |  |
| SpyM3_1509 |  | Guanine-hypoxanthine permease | -2.07 |  | -2.10 | -2.45 |
| SpyM3_1556 |  | ABC transporter ATP-binding protein | -1.78 |  |  |  |
| SpyM3_1557 | *htsC* | ABC transporter ATP-binding protein | -2.69 |  |  | -2.96 |
| SpyM3_1558 | *htsB* | Ferrichrome transport ATP-binding protein | -3.62 |  |  | -4.29 |
| SpyM3_1559 | *htsA* | Ferrichrome transport system permease | -2.53 |  |  | -2.99 |
| SpyM3_1560 | *shp* | Ferrichrome-binding protein | -2.88 |  |  | -2.64 |
| SpyM3_1561 | *shr* | Putative Fe3+-siderophore transport protein | -2.77 |  | -2.01 | -2.98 |
| SpyM3_1569 | *scrA* | PTS system, sucrose-specific IIABC | -1.51 |  |  |  |
| SpyM3_1570 | *scrB* | Sucrose-6-phosphate hydrolase |  |  | +1.61 | +1.55 |
| SpyM3_1583 |  | Hypothetical protein |  |  |  | +1.50 |
| SpyM3_1654 | *lacE* | PTS system, lactose-specific IIBC component | -1.68 |  |  |  |
| SpyM3_1656 | *lacD.2* | Tagatose-bisphosphate aldolase | -1.52 |  |  |  |
| SpyM3_1698 | *ska* | Streptokinase | +3.65 |  | +3.46 | +3.32 |
| SpyM3_1715 | *smeZ* | Enterotoxin | -1.76 |  |  |  |
| SpyM3_1717 |  | Hypothetical cytosolic protein | +5.01 |  | +2.51 | +2.70 |
| SpyM3_1718 | *dppA* | Dipeptide-binding protein | -5.20 |  |  |  |
| SpyM3_1719 | *dppB* | Dipeptide transport system permease | -7.20 |  |  | -2.01 |
| SpyM3_1720 | *dppC* | Dipeptide transport system permease | -6.11 |  |  |  |
| SpyM3_1721 | *dppD* | Dipeptide transport ATP-binding protein | -5.46 |  |  |  |
| SpyM3_1722 | *dppF* | Dipeptide transport ATP-binding protein | -6.94 |  |  | -2.03 |
| SpyM3_1737 |  | Hypothetical protein |  | +1.577 |  |  |
| SpyM3_1738 |  | Hypothetical protein |  |  | +1.60 |  |
| SpyM3_1743 |  | Hypothetical protein | +4.10 | +2.72 | +3.91 | +2.23 |
| SpyM3_1744 | *ropB* | Transcriptional regulator | +3.73 | +2.99 | +3.59 |  |
| SpyM3_1745 | *sda* | Deoxyribonuclease precursor | +2.55 | +1.56 | +2.55 |  |
| SpyM3_1759 |  | Translation initiation inhibitor | +1.50 |  | +1.66 |  |
| SpyM3_1763 |  | Dipeptidase A | +2.04 |  |  |  |
| SpyM3_1770 | *ahpC* | Peroxiredoxin | +1.61 | +1.54 | +1.64 | +1.92 |
| SpyM3_1771 | *ahpF* | Peroxiredoxin reductase (NAD(P)H) | +1.85 | +1.83 | +2.00 | +2.23 |
| SpyM3_1785 | *dexS* | Trehalose-6-phosphate hydrolase | +2.19 |  | +2.82 | +1.96 |
| SpyM3_1786 |  | PTS system, trehalose-specific IIBC | +1.80 |  | +1.97 | +1.89 |
| SpyM3_1792 |  | Acetyltransferase | -1.58 |  |  |  |
| SpyM3_1819 | *cadC* | Cadmium efflux system accessory protein | -1.53 |  |  |  |
| SpyM3_1820 |  | Hypothetical protein | -1.55 |  |  |  |
| SpyM3_1821 |  | FtsK SpoIIIE family | -1.63 |  |  |  |
| SpyM3_1822 |  | Hypothetical protein | -1.52 |  |  |  |
| SpyM3_1838 |  | Putative tRNA synthase subunit GidA | -1.57 |  |  |  |
| SpyM3_1843 |  | Transglycosylase SLT domain family protein | -1.57 | -1.52 |  |  |
| N/A^b^ | *spd1* | Streptodornase | +2.84 |  | +2.43 | +2.18 |
| N/A | *speC* | Enterotoxin | +2.87 |  | +2.48 | +2.30 |

^a^Positive numbers indicate higher gene transcript levels in indicated strains; negative numbers indicate higher gene transcript levels in wild-type strain

^b^N/A = not applicable as *spd1* and *speC* are not present in strain MGAS315 and thus do not have spyM3 designations
